# Engaging basic scientists in translational research: identifying opportunities, overcoming obstacles

**DOI:** 10.1186/1479-5876-10-72

**Published:** 2012-04-13

**Authors:** Jennifer A Hobin, Anne M Deschamps, Richard Bockman, Stanley Cohen, Paul Dechow, Charis Eng, William Galey, Mariana Morris, Sharma Prabhakar, Usha Raj, Peter Rubenstein, John A Smith, Patrick Stover, Nancy Sung, William Talman, Richard Galbraith

**Affiliations:** 1 Federation of American Societies for Experimental Biology, 9650 Rockville Pike, Bethesda, MD, 20814, USA; 2 Hospital for Special Surgery, Cornell University, 535 East 70th Street, New York, NY, 10021, USA; 3 Department of Pathology and Laboratory Medicine, New Jersey Medical Center, University of Medicine and Dentistry of New Jersey, 185 S. Orange, MSB C579, Newark, NJ, 07103-2714, USA; 4 Department of Biomedical Sciences, Baylor College of Dentistry, Texas A&M Health Science Center, 3302 Gaston Ave, Dallas, TX, 75246, USA; 5 Cleveland Clinic Main Campus, Genomic Medicine Institute, Mail code NE5, 9500 Euclid Avenue, Cleveland, OH, 44195, USA; 6 Howard Hughes Medical Institute, 4000 Jones Bridge Rd, Chevy Chase, MD, 20815, USA; 7 Department of Pharmacology - Toxicology, Wright State University, 3640 Col. Glenn Highway, Dayton, OH, 45435, USA; 8 Texas Tech University Health Science Center, 3601 4th Street, Stop 9410, Lubbock, TX, 79430, USA; 9 Pediatrics, University of Illinois at Chicago, 840 S Wood Street, M/C 856, Chicago, IL, 60612, USA; 10 Department of Biochemistry, University of Iowa - BSB, Iowa City, IA, 52242-1109, USA; 11 Department of Pathology, University of Alabama-Birmingham, W230K West Pavilion, 619 S. 19th Street, Birmingham, AL, 35249-7331, USA; 12 Division of Nutritional Sciences, Cornell University, 127 Savage Hall, Ithaca, NY, 14853, USA; 13 Burroughs Wellcome Fund, 21 T. W. Alexander Drive, Research Triangle Park, NC, 27709, USA; 14 Roy & Lucille Carver College of Medicine, University of Iowa, 200 Hawkins Drive, Iowa City, IA, 52242, USA; 15 The University of Vermont College of Medicine, Fletcher Allen HC, Baird-795, Burlington, VT, 05405, USA

**Keywords:** Basic science, Translational research, Benefits, Challenges, Recommendations

## Abstract

This report is based on the Federation of American Societies for Experimental Biology’s symposium, “Engaging basic Scientists in Translational Research: Identifying Opportunities, Overcoming Obstacles,” held in Chevy Chase, MD, March 24–25, 2011. Meeting participants examined the benefits of engaging basic scientists in translational research, the challenges to their participation in translational research, and the roles that research institutions, funding organizations, professional societies, and scientific publishers can play to address these challenges.

## Engaging basic scientists in translational research: identifying opportunities, overcoming obstacles

Basic scientists are the foundation of the biomedical research enterprise [1]. Their work is key to understanding fundamental biological processes and mechanisms of disease pathogenesis, and it has been critical to preventing, diagnosing, and treating diseases and conditions that afflict millions of people. Yet despite major advances in fundamental biology, there is widespread concern about the slow pace at which these discoveries are translated into safe and effective clinical interventions. The National Institutes of Health (NIH) estimates that 80 percent to 90 percent of potential therapeutics in preclinical testing run into problems that preclude them from advancing to the clinical trial phase.^a^ Numerous initiatives to speed translation are under way at the national and institutional levels, many of which have been aimed at providing clinical scientists with the knowledge and tools needed to translate research discoveries into improved patient care. Less attention, however, has been given to the contributions that basic scientists make to the process of translational research.

A somewhat fluid and amorphous concept, translational research is the term used to describe a spectrum of scientific investigation aimed at: 1) transferring laboratory discoveries about disease mechanisms into new methods for diagnosing, preventing, and treating disease and testing these methods in humans; 2) taking results from clinical studies into clinical practice and understanding the efficacy and dissemination of health care interventions; and 3) using the knowledge gained to improve health care policy. Scientists from many disciplines often are involved in this work, ranging from the most basic investigators in the life, chemical, physical, mathematical, engineering, and computer sciences to those in the clinic. Research across the continuum does not always proceed linearly. Often it is an iterative, bidirectional process in which insights into biological mechanisms and disease processes inform and spur new clinical interventions and, conversely, observations about the nature and progression of disease made in the course of patient care and clinical research stimulate new basic investigations. This paper focuses on the former pathway.

Although individuals trained in basic science can contribute to research at any point along this spectrum, their expertise is especially valuable during the first, or “T1,” stage. This is where an understanding of human biology, pathogenesis, and basic science methods converge to bear on the identification and characterization of disease targets, assay development, lead identification and high-throughput screening, and preclinical evaluation of potentially therapeutic compounds in molecular, cellular, and animal models. Recent research breakthroughs provide an ever-widening landscape of opportunities for basic investigators to work in these areas. These include the completion of the Human Genome Project and dramatic advances in information technology; biocomputing; high-throughput technologies for screening, identifying, and studying compounds of interest; and novel imaging capabilities. Recognizing these opportunities, funding agencies such as NIH have instituted an array of translational research programs ( Additional file 1: Table S1).

However, a number of obstacles—scientific, institutional, cultural, and policy—have limited the opportunities for basic investigators to conduct translational science. This work is best conducted through multi- and interdisciplinary collaborations, which can be difficult to establish and maintain in the current research environment—one that encourages specialization and rewards individual achievement and hypothesis-driven research. In addition, basic investigators may face inadequate funding, resources, or infrastructure for developing translational research programs. Furthermore, they often lack sufficient experience with essential methods and techniques, as well as with complex regulatory requirements, to be effective in this realm. These challenges can limit professional interest in the field and hamper the translational enterprise.

To address these issues, the Federation of American Societies for Experimental Biology (FASEB) held a symposium March 24–25, 2011, that brought together basic and clinical research trainees and scientists, department heads, and senior leaders from diverse academic research institutions; the leadership of private and public research funding organizations, pharmaceutical research organizations, and scientific societies; and scientific publishers. (For the agenda and a full list of registrants, see Additional file 2 and Additional file 3.) Meeting participants explored the benefits of engaging basic scientists in translational research, the obstacles that stand in their way, and the roles that research institutions, funders, professional societies, and scientific publishers can play to help basic scientists overcome these obstacles.

The meeting was hosted by the Howard Hughes Medical Institute (HHMI) in Chevy Chase, Maryland, and was co-sponsored by the Burroughs Wellcome Fund, the U.S. Department of Veterans Affairs, the Doris Duke Charitable Foundation, and Merck Sharp & Dohme Corp. All five organizations have a long track record of supporting fundamental science and an interest in advancing human health and the treatment of disease. To supplement information provided in the presentations, FASEB conducted a survey of meeting participants and nearly 2,000 FASEB society scientists. This report describes the major survey results (see Additional file 4), summarizes the proceedings of the meeting, and offers a practical set of recommendations for engaging basic scientists in translational research. FASEB’s goal is to foster the creation of an environment that encourages basic scientists to consider the translational potential of their work and to pursue opportunities to apply their knowledge and expertise to investigations directly relevant to improving human health and treating disease.

### The benefits of engaging in translational research

The ultimate goal of translational biomedical research is to improve human health—an outcome that benefits all of society. But participating in translational science also has more direct and immediate rewards for individual investigators and the institutions that support their work. Three teams of panelists—paired as basic scientists and senior leaders from the same university—discussed these benefits from their respective vantage points.

### Benefits to scientists

The opportunity to conduct research that has an impact on human health is a significant factor in many scientists’ decisions to participate in translational research. Nearly three-quarters of the respondents to FASEB’s survey indicated that they initially embarked on translational work because they wanted to have an impact on a particular disease or condition, and more than half wanted to have an impact on human health in general. This sentiment was echoed by Dirk Schnappinger, PhD, Associate Professor, Weill Cornell Medical College, who is working to validate new targets for the discovery of drugs to treat tuberculosis. He noted that he and his team, which includes collaborators from 13 institutions around the world, “are motivated by having an impact on global health and can attract people who share that desire.” Likewise, for Michael Dyer, PhD, an HHMI Early Career Scientist and a basic biologist studying retinal development at St. Jude Children’s Research Hospital, the realization that the tens of thousands of papers on the genetics of retinoblastoma had not yet affected the way patients with the disease are treated “fundamentally changed the direction of [his] career and the research direction of [his] lab” (see Table 1).

**Table 1:** Making a Difference: Taking the Challenges of the Clinic Back to the Laboratory

When Michael Dyer, PhD, first arrived at St. Jude as a junior faculty member nine years ago, he was the head of a basic developmental neurobiology research laboratory. One day, while deciding how to set up his laboratory space, there was a knock at his door. Two clinicians who treat retinoblastoma patients asked him to spend some time with them in the clinic. He realized that after spending five years at a major medical center and studying normal eye development and genetic mutations of the eye, he had never once met a clinician, patient, or patient’s family. He eagerly accepted the opportunity. Dyer knew that Rb gene mutations cause retinoblastoma, a rare childhood cancer with only 300 cases per year in the United States. But when he asked the clinicians if the tens of thousands of papers available on PubMed on the Rb gene and pathway had any impact on how patients are treated, they replied that they had no impact whatsoever. This surprising response led him to change the direction of his career and the research direction of his laboratory. To Dyer, the reason these basic research papers had not been translated into new therapies was obvious: there was a lack of good animal models for this disease. Researchers had tried to develop a retinoblastoma model by deleting the Rb gene from the mouse genome, but the manipulation did not cause the mouse to develop retinoblastoma. Unbeknownst to them at the time, another protein, p107, is able to compensate for Rb in the mouse. When both Rb and p107 are deleted from the mouse genome, retinoblastoma develops in about 50 percent of the animals. Using this model, he was able to test different therapeutics against the disease. In doing so, Dyer applied what he learned from his clinical colleagues about the actual course of treatment in patients (i.e., clinical reality) and tested combinations of broad-spectrum systemic 45 chemotherapeutics in a similar manner in his mice. After going through eight different combinations of drugs and comparing them to the current standard of care, Dyer and his colleagues found a combination that seemed to be better than what children currently were receiving. St. Jude started a new five-year clinical trial based on those data. Although going from nothing to a new clinical trial in about 18 months was satisfying, Dyer hoped to do better.	
	He wanted to develop a more targeted chemotherapy that could be delivered locally to the eye and result in fewer side effects. Searching for a drug target, Dyer’s team discovered that patients with retinoblastoma have increased levels of MDMX, which sequesters p53, a tumor suppressor, and leads to cell proliferation and tumor development. MDMX is amplified in 65 percent of patients and epigenetically turned on in the rest. Now having a target to go after, Dyer and colleagues developed a model in which the MDMX gene was conditionally over expressed in the mice that develop retinoblastoma. This resulted in a much more aggressive disease in these animals—providing a new model to test chemotherapeutics. Not wanting to rely solely on a genetic model, they also developed a “xenographic” mouse model in which human tumor cells are transplanted to an eye of an immunocompromised mouse. This results in virtually 100 percent engraftment with tumors expressing high levels of MDMX. Collaborations with his chemical biology colleagues, who were able to synthesize an MDMX inhibitor, and clinicians, who provided medical information and tumor specimens, enabled Dyer to begin a preclinical trial focused on inhibiting MDMX. Dyer believes that the process by which basic investigators can translate their discoveries into clinical applications could be emulated at most major medical or academic research centers. The building blocks important for driving translation, such as strong basic science departments, animal research facilities, pharmacology and pathology departments, and clinical trial support are already in place. He also noted that some translational work can be done with relatively few resources if one acts creatively. He received no support from St. Jude to fund these studies directly. Rather, Dyer built on a one-year pilot grant of $50,000 and cobbled the rest together through other sources. The challenge, he said, is changing the culture. Emphasizing the value of translational research to both basic and clinical scientists, encouraging communications between basic and clinical scientists, mentoring, and understanding the clinical reality of disease progression and therapeutic interventions are all important for increasing participation in translational research and ultimately improving human health.

Thomas Tuschl, PhD, Associate Professor and Head of the Laboratory for RNA Molecular Biology at The Rockefeller University, also wanted to apply his science to the study of disease; partnering with clinicians helped him to do that. Trained as a basic scientist, he obtained his doctoral degree in chemistry, conducted postdoctoral work in biochemistry, and then became interested in the therapeutic applications for gene silencing through the discovery of sRNAs and microRNAs as regulators of gene expression. His current work is focused on defining the molecular pathology leading to specific diseases and developing targeted approaches suitable for therapeutic interventions.

Tuschl had developed an appreciation for translational science and its challenges while working with the pharmaceutical industry. As a cofounder and scientific advisor to Alnylam Pharmaceuticals and Regulus Therapeutics, he learned a great deal about genetic diseases, the molecular causes underlying disease, and validation of drug targets, and he started to look for translational research opportunities that were within reach of his academic laboratory. His commitment to the field increased when a group of highly motivated Clinical Scholars joined his laboratory. These clinicians were attracted to his work primarily because of its specialized methods for characterizing small regulatory RNAs and their targets, but their projects rapidly expanded and touched on many aspects of gene regulation. He had not anticipated the strong interest that the clinical investigators would have in basic science and new methodologies, and he found that pairing each clinician with a basic investigator resulted in a considerable amount of synergy between the two.

Without the expertise that the clinicians brought to his laboratory, Tuschl says he might have never understood how to operate and devise experiments relevant to translational medicine. By working with human tissue samples, observing the variation among individuals and in sample collection, following the clinicians’ progress at the bench, and accessing their data, he learned how to adapt his methods to study hundreds of patient and control samples. In return, the scholars have found it helpful to train with a basic scientist, which offered them a completely new strategy for understanding biological phenomena.

For basic biologists, engaging in translational research has benefits that go beyond contributing to understanding and treating human disease. It helped Leslie Vosshall, PhD, Robin Chemers Neustein Professor and Head of the Laboratory of Neurogenetics and Behavior at The Rockefeller University, to learn more about her field. Trained as a basic biomedical researcher, Vosshall used insects to study smell for many years before turning to people where, she said, “all the interesting action is.” Today, she studies how humans discriminate among smells, how genetic variation in humans alters how they perceive odors, and how mosquitoes react to our smell. Her laboratory is one of the biggest users of the Rockefeller outpatient clinic and has run upwards of 1,500 volunteers through these olfaction studies.

Pursuing translational research also had benefits for Daniel Wagner, PhD, Assistant Professor, Department of Biochemistry and Cell Biology at Rice University. A basic developmental biologist working in zebrafish, Wagner embarked on a collaborative project with a physicist after encountering him reading one of Wagner’s posters in the hallway. The physicist was working on a theranostic (therapeutic-diagnostic) application for plasmonic nanobubbles, in which gold particles are excited by short laser pulses to generate transient vapor bubbles. The biologist and the physicist joined up to test the concept that these nanobubbles could be used to identify cells based on their expression of specific cell surface molecules and to destroy specific cells in a zebrafish. The team used these nanobubbles targeted to the EGF receptor and was able to destroy prostate cancer cells within a host embryo. This was the first step—proof of principle—that the theranostic method can be used in vivo as a potential diagnostic tool and ultimately for targeted therapy.

“As I saw the group that was being assembled around this plasmonic nanobubble project, I really began to think that there were some serious benefits for me, as a basic scientist, and the potential to move this technology forward and ultimately translate it into the clinic,” said Wagner. Among the benefits he cited were participation in a quickly moving multi-investigator project that produced novel results; exposure to new fields and methods leading to new projects; and rapid publication rate. Indeed, Wagner noted that three papers were generated from the nanobubble project in the year preceding the meeting, equal to the output of all of his other projects combined.

In sum, participation in translational research benefits basic scientists by:

· Enabling them to contribute to the understanding of human health and disease and to participate in the development of solutions to medical and public health problems, which can be a source of intellectual inspiration and stimulation.

· Providing opportunities to develop their own science and learn new methods, which can lead to new projects, fresh directions for existing projects, and faster publication rates.

· Promoting collaborations with clinicians, clinical researchers, and basic investigators in other disciplines, which can ignite shared passion, provide exposure to new areas of science, and generate new ideas.

· Providing opportunities to mentor clinical colleagues in basic science methods.

### Benefits to institutions

Institutions also benefit from creating an environment conductive to translational science. Their efforts help their scientists by facilitating access to resources that they may not have had before and connecting them to collaborators with whom they share research interests, but otherwise may not have met. These synergies move research forward, benefitting the individual and his or her institution. For example, at The Rockefeller University, Vosshall noted that in order to carry out her studies on the sense of smell, she increasingly has to rely on the resources that are funded by Rockefeller’s CTSA. Support for statistical work comes from its biostatisticians, and its facilitation office has helped shepherd protocols from initial conceptualization to final approval. She has had to lean heavily on the clinical staffing in the outpatient clinic, and the Institutional Review Board (IRB) has helped improve the quality of her protocols. Working with Rockefeller’s hospital, she was able to obtain access to the darkened space that she needed for her own research by acquiring repurposed rooms that had been used for sleep studies. Her work would not have been possible without these resources.

A focus on translational research also benefits trainees and early career scientists. It provides important training opportunities for graduate students and postdoctorates and may help to attract new talent into biomedical research. Cindy Farach-Carson, PhD, Vice Provost for Translational BioScience, Rice University, noted that translational research appeals to today’s generation of university students—many of whom want to work on “big, real world problems.”

Farach-Carson also emphasized that establishing the infrastructure to conduct translational research provides opportunities for all of an institution’s investigators—not just those working on bench-to-bedside projects. The collaboration necessary for conducting this kind of work involves sharing expensive equipment, forming equipment cores, and developing policies for sharing, which can work to everyone’s advantage. For example, Rice’s $9 million supercomputer from IBM came with the condition that it be shared in an affordable way with the Texas Medical Center and others outside the center.

In addition to benefiting their scholars, investing in translational research has a positive impact on institutions as a whole. Fostering an environment conducive to translation advances the core missions of biomedical research institutions, stated Barry Coller, MD, Vice President for Medical Affairs and Physician-in-Chief, The Rockefeller University Medical Center. Successes in the development of new drugs, devices, and procedures attract patients who want to benefit from cutting-edge care, and they attract funding from donors with an interest in supporting promising work that could lead to new treatments.

Scott Weir, PharmD, PhD, Director, Institute for Advancing Medical Innovation, University of Kansas Cancer Center, described the evolution of the extensive network of partners that formed around efforts to establish a center of excellence for cancer at his institution. Establishing a National Cancer Institute-designated cancer center became a university, state, and regional priority. The center’s goal was to capitalize on the strengths of the university and on regional assets to become a national leader in the discovery and advancement to Phase I of promising anticancer agents.

Before the center was started, the University of Kansas had one of the top schools of pharmacy in the United States and strong leadership in medicinal and pharmaceutical chemistry. It also had conducted research that led to the commercialization of several drug products. But none of those products had been tested in patients at the university’s medical center, which is located 42 miles away from the School of Pharmacy. Additionally, its cancer biology program was in its infancy and did not have a rich, potential source of novel cancer drug targets. To achieve its goal of becoming one of the top university-based cancer centers in bench-to-bedside translation, the university identified the need to establish translational research competencies and capabilities. Pharmaceutical industry veterans were recruited to establish best practices. In addition to working with the University of Kansas cancer biologists, efforts to establish partnerships with industry, academia, government, and disease philanthropy collaborators were initiated to gain access to novel drug targets.

The university has refined its role in cancer therapeutics. With a focus on unmet medical needs such as rare and neglected diseases and pediatrics, it has established translational research processes to discover and develop novel drugs as well as identify new uses for approved and abandoned drugs. It has integrated and even rebuilt core facilities, developed a strategy for handling technology transfer, put proof-of-concept funding strategies in place, and formed multidisciplinary and multi-organizational teams managed by industry-experienced project managers. The center also invested heavily in project managers who oversee all aspects of translation, freeing up investigators to focus on the science, and it is looking beyond its own walls for promising ideas to move drug development forward.

By demonstrating successes in translational research, the university was able to attract top-notch cancer researchers who are interested in drug discovery and development. It has recruited more than 230 years of pharmaceutical experience over the past five years, from investigators pursuing target validation to those doing clinical testing. Its collaborations have brought in a broad array of new funding sources to support additional collaborations, including those involving government and philanthropic organizations and those related to state economic development initiatives. The center brought three new therapies to the clinic in 2009, moved one forward in 2010, and brought five more to the clinic in 2011.

In sum, institutions benefit from fostering translational research programs by:

· Connecting their investigators to resources and collaborators and facilitating affordable access to expensive shared equipment and facilities.

· Providing unique training opportunities for undergraduates, graduate students, and postdoctorates and encouraging new talent to enter biomedical research.

· Promoting the development of new drugs, devices, and other medical interventions, which advances their biomedical research missions, attracts patients, and enhances their status.

· Gaining access to new funding streams supporting both institutional and individual projects.

· Attracting public-private partnerships and leveraging federal and nonfederal resources.

### Challenges to engaging basic scientists in translational research

Meeting participants noted that numerous challenges can discourage or prevent basic investigators from participating in translational science. These include differences in culture and mindset between basic and clinical researchers, insufficient and/or nonsupportive infrastructure, difficulty developing and sustaining collaborations, inadequate training, insufficient funding, and lack of other incentives and rewards.

### Culture and mindset

Basic scientists may see their controlled, hypothesis-driven research as more rigorous than the goal-directed or descriptive research often conducted with humans in clinical research settings. On the other hand, clinical scientists may view their work as superior insofar as it has greater relevance to human health and disease. These perceived differences not only inhibit collaborations between the two groups, but also they may discourage basic investigators from pursuing translational projects (see Table 2). Even when basic scientists are open to translation, they do not necessarily speak the same language as their clinical investigator counterparts because of differences in training and experience.

**Table 2:** Going It Alone—Sometimes the Translational Scientist Is His or Her Own Best Ally

Daria Mochly-Rosen, PhD, Professor and Senior Associate Dean for Research and Director of SPARK, Stanford University’s Translational Research Program, is a protein chemist conducting translational research. Early in her career, she designed rational inhibitors that could turn off heart cell enzymes one at a time and discovered enzymes that could change the rate at which heart cells in culture beat. She thought this was an important finding that would be of interest to the heart research community, but when she presented her work at a scientific meeting, she found the audience to be disinterested in heart rate regulation. Clinicians, she had been told, already had ways of managing heart rate; they were concerned with problems such as cardiac ischemia. When she brought her idea directly to industry, company after company turned her away. She later realized that there were good reasons for this: while her work was attractive from a basic research point of view, the barriers to executing it in patients were huge, and many steps had to be completed before reaching that goal. Mochly-Rosen reached out to her colleagues for assistance, but rather than finding support, she was discouraged from pursuing this line of inquiry and from working with industry. Translational research, she was told, is not intellectually challenging, worthwhile, or good for her career. “Career progress in academia is measured by how many papers are published and how much grant funding is received rather than, for example, attempting to produce a new drug,” she said.	
Following the advice of a colleague, she invited into her laboratory a physician who wanted to learn basic research and from whom she could learn how to study more clinically relevant problems. Her work eventually led to the discovery of an inhibitor that when administered after a heart attack dramatically reduces heart damage by 70 percent and prevents subsequent heart failure, a finding that was demonstrated in mice, rats, guinea pigs, rabbits, and pigs. Patents were written and the results published. Yet no one was interested in her findings. Why would this not be useful in patients, she thought? But she persevered, formed her own company, KAI Pharmaceuticals, and spent a year as its Chief Scientific Officer. She helped write an Investigational New Drug Application and launched a clinical trial. “Unlike my training in academia, where questions led to the research, in industry I learned to think about the final product and work backwards, to identify what research needs to support such a product,” said Mochly-Rosen. The process was humbling, but also gratifying. “There is nothing more rewarding than [treating] the first patient…or when the trial is finished, looking at the data. It’s really a true manifestation of what basic research should eventually lead to.”	

Noting that she comes from a family of clinical physicians, F. Nina Papavasiliou, Associate Professor and Head of the Laboratory of Lymphocyte Biology, The Rockefeller University, stated that “There has been, always, a little bit of a mistrust from my end [as] the basic scientist. I always felt that clinical research was either not rigorous enough or boring. And my parents and siblings always felt that basic research was not really useful to them when they would see, maybe, the outcome of it in their practice 10, 20, or 30 years later—if they’re lucky.”

Institutions where translational research flourishes have had to focus on overcoming the cultural differences between basic and clinical scientists. They do this by creating opportunities for these investigators to share perspectives, see the value in each other’s work, and embrace both hypothesis testing and goal-oriented research. Philip Haydon, PhD, Professor and Chair, Department of Neuroscience, Tufts University School of Medicine, said he was brought to Tufts to change the culture. When he moved into the university’s neuroscience department, there was a focus on synaptic physiology, but very little of the work had any translational thrust. He was faced with the challenge of identifying translational opportunities for faculty engaged in rigorous, basic work and helping to move them along that path.

One way Haydon is trying to do this is by developing three faculty clusters that act much like magnets. These clusters (in epilepsy, depression, and neurodegeneration) are open to trainees, as well as faculty, and they meet every two to three weeks for lunch to hear talks about their work or discuss a particular topic. As a result, people who had no interest in diseases are now gaining interest. Haydon has observed that faculty members who are surrounded by others with a translational focus are more likely to develop the interests and skills needed to adapt. Indeed, Papavasiliou noted that having a clinical fellow in her laboratory “provided a little dose of humility on both ends and fostered the idea that we can, in fact, both make an impact quickly while learning from clinical studies. From our clinical samples, we’ve learned a lot about transcriptome editing that we probably wouldn’t have trusted had it come from mouse studies alone.”

### Insufficient infrastructure to develop clinical protocols, facilitate exchanges, and enable handoffs

Many basic scientists (and some clinical scientists) find the pathway to clinical application filled with unfamiliar requirements and administrative procedures. Translational research involving human subjects may require IRB and Privacy Board approval, reporting to data safety monitoring boards, clinical trials registration, and specialized patient recruitment techniques. Working with industry adds a level of complexity, because conflict of interest disclosures and data use and intellectual property agreements must be drafted. As a result, carrying out a clinical protocol can require more steps and time than a laboratory-based protocol. Basic investigators usually do not have experience navigating these requirements, and their universities may or not have the expertise and infrastructure to help them.

Some institutions have been proactive in addressing these needs. The Institute for Advancing Medical Innovation at the University of Kansas Cancer Center forms project management teams around technologies ripe for translation. These teams provide investigators with the expertise they need in areas such as drug discovery and development, industrial and philanthropic partnering, technology transfer, and medicinal chemistry. This support ensures that investigators can focus on their area of expertise: the science.

Rockefeller also has developed support systems for translational investigators: it facilitates the development of clinical protocols by providing expertise in the protection of human subjects, subject recruitment, compliance with trial registration, and protocol monitoring and auditing, among other areas. The institution employs research nurses trained in care delivery, and medical personnel are available to participate in protocols led by a PhD. Clinical research coordinators, who play critical roles in regulatory compliance and data integrity, are also available to any scientist who needs them, as is a customized, comprehensive information technology platform that allows for conducting protocols.

Rice University is developing a researcher’s toolbox—a website where everything that is needed to embark on translational research projects can be found. For example, to make it easier for investigators to collaborate, Rice is creating templates for drafting research agreements, establishing protocols for sharing IRBs, and developing animal care protocols that would allow the movement of experimental subjects and materials between performance sites. It also is working with area biotechnology companies to build a translational and commercial component into the toolbox. These support systems are critical for helping basic scientists move their discoveries through the translational research pipeline to commercialization.

### Developing, establishing, and sustaining collaborative research

A successful translational research environment requires many different people, trained across many disciplines, working within a structure that promotes interaction between those who have clinical understanding of human health and disease and those who have training and expertise in fundamental biology, the molecular mechanisms of disease, and the use of animal models. “To solve many of the biggest challenges in health care,” said Gail Cassell, PhD, Visiting Professor, Department of Global Health and Social Medicine, Harvard Medical School, and Vice President of TB Drug Discovery, Infectious Disease Research Institute in Seattle, “It is absolutely essential…that we have basic scientists working along with the clinical scientists. And it is essential that we have the public and the private sectors working together from the very outset.” Establishing these collaborations is challenging, however. Academic departments tend to operate in silos, and basic scientists might not even work on the same campus as clinical researchers, limiting interactions that might spark translational research projects and making it difficult to obtain assistance and mentorship. It can be even more difficult for academic and government researchers to forge relationships with industry partners, because concerns about perceived or real conflicts of interest can have a chilling effect on collaborations.

Funding organizations and institutions have been working to bring basic and clinical—and academic and industrial scientists—together through investments in translational research resources and infrastructure. NIH’s CTSA program has been an important driver in encouraging institutions to foster these collaborations, and institutions are experimenting with a variety of approaches. The University of California, San Francisco, for example, is trying to minimize geographic distances between academic and industrial researchers by co-locating their laboratory space as it did in a recently forged partnership with Pfizer. Ensuring that core resources are available across departments is another strategy. Providing access to statistical centers, biorepositories, or datasets, for example, can foster cross-fertilization. Institutions have started mentoring programs through which basic scientists mentor clinical researchers or vice versa, while others have convened networking events and regular seminars or workshops that bring diverse disciplines together. Some academic medical centers have begun asking basic scientists to give presentations during grand rounds. The key to creating an environment that facilitates collaboration is to increase exposure to the other research cultures through closer proximity. Cassell also noted that investigators who have an idea that they want to translate “should engage industry partners as soon as possible in the process. All too often, investigators, whether they are in government or in academia, will wait until they have a solidified plan, a strategy, and then go seek funding from the industrial partner. Another equally important mistake is that industry is looked upon as only potential funders and not scientific collaborators. The most value industry can offer is unique expertise.”

### Training basic investigators in translational research

There always have been basic scientists who are adept at transforming their research discoveries into clinical advances. But as the pressure to accelerate the pace of translation builds, so does the need for a cadre of basic investigators with the motivation and skills to apply their work directly to the improvement of human health. According to Coller, there are three skills that translational investigators must learn. The first is the ability to define a health need with the same precision as a basic science hypothesis. The second is to understand how to develop an inexpensive, robust, high-throughput assay applicable to humans. The third skill is to be able to conceptualize a pathway to regulatory approval or clinical adoption. Although significant strides have been made in developing translational research training programs that teach these and other relevant skills, inadequate experiential and didactic training remain a barrier for many basic investigators.

Whereas clinical examples are integrated into the basic science coursework many medical students are required to take, graduate students in basic research programs typically are not exposed to clinical cases. Furthermore, doctoral students often do not receive instruction in pathobiology and pathophysiology, which are key to understanding the mechanisms of disease and the disease relevance of their work. Indeed, Farach-Carson of Rice University noted that graduate students have less contact with basic concepts of medicine than in the past. Another participant noted that “trainees frequently are more knowledgeable about molecules and structural biology than about the systems in which they function.” These limitations, combined with little to no opportunity to interact with clinical researchers or patient populations, mean that many basic scientists may not think about the science that they do in the context of human health and disease, and they may not appreciate the unmet clinical needs or the clinical context in which potential interventions would operate. In addition, depending on an investigator’s area of interest and his or her particular research, there may be a need to develop familiarity with topics that are not generally covered in basic science training programs. These may include an understanding of disciplines such as biostatistics, pharmacology and toxicology, biomedical informatics, clinical research design, and regulatory processes.

The structure of basic research training at some institutions may itself present a barrier to acquiring the requisite knowledge and skills. Translational research is best conducted by interdisciplinary research teams. Basic biomedical science graduate students, however, typically choose a discipline, a department, and even a research topic early in their education. They generally are trained on research grants that support the work of a small area of biology, and their training is designed to prepare them for a career in individual investigator-initiated research in a very narrow field. Thus, not only may the concept of team-based, interdisciplinary research feel foreign to them, but also they may not have the training to pursue such collaborations successfully.

Narrowly focused training is not unique to basic investigators. While basic science education may not prepare trainees to pursue the medical applications of their discoveries, medical education tends not to require sufficient competency in basic science research to allow the physician to recognize or facilitate the application of scientific discoveries to medical or public health practice. One panelist noted that medical students typically are not told how they can be a part of the discovery process. Communication and collaboration among basic investigators, physician-scientists, and clinical practitioners are key to advancing translation, and more should be done to bridge this gap during training.

Biomedical research funding organizations recognize that this divide exists, and some have stepped in to help close it. HHMI’s Med into Grad initiative supports and encourages graduate schools to integrate medical knowledge into their doctoral training. The program’s goal is to produce researchers who will recognize which biological problems are of the greatest clinical relevance, have the knowledge and skills necessary to facilitate the translation of new biological knowledge into tools to improve human health, and be able to create fruitful research partnerships with physicians. To do this, HHMI has awarded 36 four-year grants totaling $26 million to institutions offering a doctoral degree in basic biomedical sciences. These awards have been used to enhance existing graduate programs and initiate new ones.

Recognizing that there are multiple ways to achieve its goal, HHMI has allowed institutions the flexibility to decide what educational components to include and how their programs will be structured. For example, Mary Estes, PhD, Professor, Department of Molecular Virology and Microbiology, Baylor College of Medicine, described how her institution has put its Med into Grad award to use. Baylor’s Translational Biology and Molecular Medicine Graduate program provides doctoral students with rigorous training in the basic biomedical sciences that prepares them for participation in clinical and translational research. Course work includes genetics, human physiology, immunology, molecular methods, method and logic of translational research, cellular and molecular biology of disease, gene regulation, animal models, pathophysiology, biostatistics for translational research, ethics, conduct and practical aspects of clinical research, and a bench-to-beside journal club. In the second year, students embark on their thesis research and begin clinical project training targeted at specific areas, including cancer, digestive system disorders, infectious disease, and reproductive disorders. They also receive dual mentorship by a basic scientist and a clinical scientist throughout their graduate training. The clinical mentor interacts with students in clinics, inpatient hospital rounds, and medical or research conferences where students observe clinical care and observe or participate in clinical research. The clinical mentor also is a member of the thesis committee and in many cases helps design a translational aim of the student’s research project. This program, like many others sponsored by HHMI, trains PhDs to be effective translational researchers and has forged numerous collaborations between participating PhD and MD faculty.

NIH’s CTSA program also is beginning to shape translational research training of basic biomedical science investigators. Institutions receiving CTSA funding are expected to provide research, education, training, and career development in clinical and translational sciences. With funding from the program, The Rockefeller University initiated a master’s program in clinical and translational science. It also has developed a one-year certificate program to provide basic science trainees with an introduction to the principles and practice of clinical and translational research and the opportunity to develop a translational research protocol. In addition to developing their own training programs, CTSA institutions have been working together to develop best practices for clinical and translational research training. For instance, the CTSA consortium created a set of core competencies that includes 14 thematic areas that should shape the training experiences of junior investigators. The competencies define the skills, attributes, and knowledge needed for this type of research and can serve as a guide for anyone considering the development of a translational curriculum at multiple graduate levels.

Although national award competitions have catalyzed the establishment of training programs, some institutions, recognizing the value of translational science, have embarked on their own initiatives. The MD Anderson Cancer Center’s Translational Research in Multi-Disciplinary Program (TRIUMPH), for example, is unique in its focus on training postdoctoral- level scientists.

In addition to providing training at the graduate and postgraduate level, some meeting participants suggested that it may be useful to open the possibilities of translational research to undergraduates and even high school students. This may present a unique opportunity, because students at these levels might more readily embrace translational research as a career path, not yet having formed narrower conceptions of their future careers. Baylor, for example, offers a summer program for undergraduates, in which students may work in translational research. Estes pointed out that several of the students in its Translational Biology and Molecular Medicine Graduate Program initially were exposed to translational science through that program. Similar programs for undergraduate students have been established at MD Anderson Cancer Center and The Ohio State University.

### Availability and types of funding

Many of the translational research success stories described during the meeting had one thing in common—a component that involved the creative piecing together of resources from many places. Despite national efforts to encourage translational science, some meeting participants lamented the lack of support for individual investigator-initiated projects in this area. And, while making a move to translational research can open up new funding streams for some basic scientists, others find themselves at a disadvantage in applying for grants to support that work. They note that study sections do not always have the expertise appropriate for reviewing translational protocols. Reviewers may not appreciate the merits of applied or translational work, and they may be more sensitive to the strengths of basic, hypothesis-driven proposals. Moreover, investigators who are able to acquire funding note that it is often insufficient to cover the full cost of a project. At least one presenter reported that he had to siphon money away from existing projects to support this work.

Mechanisms of support for trainees may not be optimal for encouraging the pursuit of translational research projects. Although there are programs designed to provide training in translation, as described earlier, these opportunities are limited. The vast majority of postdoctoral fellows supported by NIH are funded through individual investigator-initiated grants, and they are required to focus on the research for which the grant was awarded. This limits opportunities to expand their projects into the translational space or to work on collaborative projects that may ultimately spark new research directions. It also may create a certain amount of tunnel vision with regard to their research interests.

Research funders have taken different approaches to supporting the participation of basic scientists in translational research. In addition to its investigator-initiated research grant mechanisms, NIH provides funding through targeted programs to encourage research in specific areas of translation. Its CTSA program, while not providing direct support to scientists, has catalyzed institutional efforts to engage and support basic investigators and trainees. The agency also has mechanisms to provide investigators with access to translational research resources and equipment. The U.S. Department of Veterans Affairs has a small intramural research program. Although about 60 percent of its investigators are clinicians, it supports a fair number of basic investigators and has the flexibility to convene teams of basic and clinical scientists around translational research projects.

Private support is more limited, but it does exist. Some foundations support basic investigators conducting fundamental research in areas relevant to a disease for which they want to provide research funding. Other organizations are less interested in funding a particular project than they are an investigator who is likely to make an impact. HHMI’s investigator program, for example, focuses on “people not projects,” said Dennis McKearin, PhD, HHMI Senior Scientific Officer. “By giving basic investigators flexible budgets large enough to explore new areas [and] with low administrative burdens,” they have the ability to move good ideas from the bench to the bedside. Participants generally agreed that a diversity of approaches, including support for infrastructure and investigators and for targeted and open-ended research, would be important to engaging basic investigators and advancing bench-to-bedside translation.

### Lack of recognition and incentives

Academic tenure and promotion policies are one area in which incentives may not be optimal for encouraging basic investigators to develop a translational research program. Tenure-track faculty are required to bring in research funding and make an identifiable intellectual contribution to their fields to gain tenure and promotions, and these contributions are usually measured in research grants and publications. Moving a discovery through the translational research pipeline often involves team science that may require as many as 30 or 40 collaborators. Tenure, promotion, and appointment committees are challenged to evaluate the importance and impact of the individual contribution a single faculty member makes in the context of a multi-investigator translational project. The existing paradigms used to evaluate basic science faculty, including numbers of grants and publications, are not always sufficient to capture the merits of faculty contributions to the translational research enterprise.

Tenure and promotion committees also may undervalue the contributions of scientists who bring technological expertise to collaborations—such as mass spectrometry experts—even if that technology was key to the experiments and resulting publications. Moreover, compared to basic science studies, it often takes longer to bring clinical and translational research projects to fruition, resulting in a longer duration between publications, which may disadvantage faculty during their evaluations. This problem is magnified by a lack of understanding of translational science that can prevail among multicollegiate faculty serving on tenure committees in university senates when clear guidelines for evaluating collaborative and/or translational research are absent or marginalized. Recognizing the shift toward team-based, interdisciplinary, translational science, some institutions have started to explore changes in their tenure and promotions policies and processes to ensure that investigators are recognized for their research contributions.

Adding to these difficulties are challenges scientists face in publishing their translational work. Like study sections and basic science promotion committees, editorial boards and reviewers may place a higher value on hypothesis-driven basic science, making it difficult to publish translational research. Moreover, journals may not have a mechanism to acknowledge the contributions that each member of a research team makes to the published work. Although journals devoted to translational research may address these concerns, only a few of them exist.

Many of the concerns cited by meeting participants were echoed by respondents to FASEB’s translational research survey. More than 50 percent of survey respondents found it difficult to obtain sufficient funding for translational research, navigate human subjects protections regulations, obtain the skills necessary for developing drugs and devices, and access clinical samples. Obtaining didactic and experiential training, being recognized for team-based research, earning tenure and promotions, as well as communicating across disciplines and identifying collaborators also were cited by many respondents as being difficult.

The survey also revealed that translational investigators found it less difficult to overcome certain barriers than respondents who had not participated in translational research perceived it to be. Compared to respondents without translational research experience, translational investigators found it less difficult to obtain clinical samples and other necessary resources; obtain guidance, mentorship, skills, and training; identify collaborators; and identify the health relevance of their research. It may be that establishing a translational research program is less difficult than investigators perceive it to be or that those who encounter difficulties ultimately decide not to pursue this line of inquiry. Whatever the explanation, reducing the barriers will likely encourage more basic scientists to explore the translational potential of their work.

### Recommendations

There is much that investigators, institutions, professional societies, publishers, and funding institutions can do to support and facilitate the involvement of basic scientists in translational research: creating more training opportunities, reforming recognition and reward policies, fostering collaborations, and providing diverse and flexible forms of financial support will help. (See Additional file 5 for a list of resources.) But in addition to creating optimal research and training environments, we must ensure that there are basic investigators who want to pursue translational science. The research community should explore ways to spark interest in translation among trainees and established investigators who may not have considered how they could apply their own interest and expertise in basic biological science to meet public health needs. All of these efforts will require leaders who are willing to take risks, create new funding models and reward systems, and provide enhanced environments for science education, training, and collaboration.

### Overarching recommendations

· Funding agencies should continue to support basic science and ensure a deep and broad reservoir of new knowledge from which translational research can flourish.

· Additional funding should be provided for both individual and team-based investigator-initiated translational research grants, beyond the support for translational research infrastructure currently available.

· Institutions, scientific publishers, professional societies, and individual scientists should encourage and facilitate a “cultural shift” toward greater collaboration, cooperation, communication, and respect among basic and clinical scientists. The unique roles of both clinical and basic scientists in translational research must be valued and their contributions to each other’s research programs recognized, and this commensal approach must be modeled for the next generation of scientists.

· Institutions should examine promotion and tenure processes to ensure that the expectations of basic and clinical scientists engaged in translational research are clearly articulated and that their research contributions are quantified, evaluated, and recognized relative to the established expectations

· Institutions and professional societies should provide environments and opportunities that facilitate interactions between basic and clinical researchers to spark collaborative activities that will expedite the translation of discoveries to application.

### Recommendations to promote interest, education, and training in translational science

The first steps in facilitating the participation of basic scientists in translational research are to spark their interest in advancing the health applications of their work and help them cultivate their ability to do so. High school and undergraduate students should be made aware of potential career paths in translation, interested graduate and postdoctoral scientists should be engaged early in their careers, and all investigators should have opportunities to acquire relevant experiential and didactic training.

It is recommended that institutions:

· Provide didactic coursework, case-based learning, and seminars that cover a variety of disease topics and clinical issues.

· Incorporate pathobiology and pathophysiology courses into basic science training programs—fields that are especially important foundations for understanding human health and disease processes.

· Provide opportunities and release time for basic scientists and trainees to acquire clinical experiences that will help put basic research in context. This can include opportunities for basic scientists to participate in ward rounds; attend clinics; observe clinical procedures, autopsies, and surgeries; and/or take minisabbaticals to acquire extended training in and understanding of the experimental paradigms possible in humans.

· Facilitate training in the wide range of disciplines and skills needed to conduct translational research effectively.

It is recommended that professional societies:

· Explore a variety of approaches to spark interest, education, and training in translational research through professional meetings and other activities, including:

· Highlighting translational research at meetings by calling out these sessions in meeting materials or creating a translational research track for poster presentations and talks.

· Establishing networking opportunities for basic and clinical investigators and trainees with similar research interests.

· Offering workshops that provide basic and clinical scientists and trainees with perspectives on translational research and practical tools for establishing translational research programs.

· Providing information about translational research training and career opportunities to students at scientific meetings, including student-oriented meetings.

It is recommended that funding organizations:

· Establish and evaluate translational research training mechanisms for basic investigators and trainees.

· Encourage and support the mentoring of basic scientists and trainees in translational research, including mentoring by clinical and translational scientists and by clinicians.

It is recommended that individual scientists:

· Learn to define a health problem with the same precision as a basic science hypothesis.

· Actively seek and be open to the perspectives of scientists from different disciplines.

· Seek opportunities and funding to acquire translational research training and mentorship, including through course work, seminars, and workshops; rotations in or collaborations with clinical research laboratories; and participation in clinical experiences.

It is recommended that institutions, funding organizations, and professional societies work together to:

· Establish and/or facilitate the establishment of summer research experiences for high school and undergraduate students that provide exposure to translational research.

### Recommendations to promote access to translational research collaborators and resources

If basic investigators are to play a meaningful role in translating their discoveries into health applications, then they must have opportunities to interact with clinical investigators, clinicians, and industry partners as well as investigators from other disciplines. They also need access to the tools and resources necessary to conduct translational science.

It is recommended that institutions, funders, and professional societies:

· Facilitate interaction and collaboration among basic and clinical scientists and clinicians across sectors and disciplines, including among researchers in the life and physical sciences and mathematics through a variety of approaches, including:

· Creating training and funding mechanisms that encourage interdisciplinary or multisector partnerships.

· Establishing or supporting seminars and workshops as well as networking opportunities (e.g., research “speed dating”) that bring scientists and clinicians from different disciplines and sectors together.

· Creating databases that facilitate the identification of potential collaborators.

· Facilitate access to the research and development resources and tools that basic scientists need to conduct translational research. For example,

· Institutions and funders should support centers or programs that provide translational research services, including clinical support and expertise in developing technology transfer and data sharing agreements, writing IRB protocols, recruiting patients, and executing clinical trials.

· Professional societies should publicize the availability of national resources and funding opportunities and provide training and information to help members compete successfully for these opportunities.

### Recommendations to recognize and reward translational scientists

Incentives are needed to encourage and support basic scientists interested in pursuing translational research. Institutional retention, promotion, and tenure policies and publishing policies should be modified to ensure that investigators are recognized and rewarded for the contributions that they make to translational science.

It is recommended that institutions:

· Enable investigators to contribute to basic, clinical, and translational science and recognize that evaluation in different areas may require enlisting the expertise of faculty from other than the investigator’s primary department.

· Ensure that promotion and tenure guidelines are clear with regard to how investigators will be evaluated for contributions they make to interdisciplinary and team research.

· Carefully consider the composition of tenure review committees so that nontraditional—but still rigorous—scholarship is recognized.

· Consider extending the tenure clock to accommodate scientists involved in translational studies involving human subjects, which often take longer to complete and publish than studies that do not involve human subjects.

· Consider the impact of a basic investigator’s work, not just the quality or quantity of publications, when evaluating his or her publication records.

It is recommended that scientific publishers and editors:

· Ensure that the roles of authors are clearly articulated in publications.

· Highlight the potential contributions of basic research findings to public health by publishing articles or editorials that illustrate the implications a particular finding has for translation.

· Encourage high-impact journals to embrace translational studies (with the caveat that there is still a need for specialty journals that focus on translational research).

It is recommended that individual scientists:

· Be aware of tenure expectations, clearly articulate their career goals, and negotiate department concurrence. They should define the intellectual space or spaces in which they are going to be leaders, especially if translational science is to be important in achieving tenure.

· Seek reviewers who understand and value the contributions they make to translational research when soliciting tenure review letters.

### Recommendations for funding organizations

Public and private funders of biomedical research play a key role in encouraging the entry of basic scientists into translational research. Funders provide direct research support, access to research resources and equipment, and support for the development of training programs. Funding policies and incentives also can motivate the institutional changes needed to support basic investigators. Together, organizations that support translational research have a wealth of information about the efficacy of various programs and policies that could be used to optimize the enterprise going forward.

To attract basic scientists to translational research, it is recommended that funding agencies:

· Recognize milestone and outcome-driven projects as well as those aimed at open-ended inquiry when making funding decisions.

· Fund translational research projects that engage investigators across disciplines.

· Provide supplements to basic research grants that allow investigators to extend basic research findings into the translational research domain. Similarly, provide supplements to clinical investigators to conduct laboratory work based on clinical findings.

· Provide sustained support for translational research infrastructure, commercialization centers, and research training programs.

· Sponsor meetings and other activities to bring groups of researchers together to interact and learn from one another.

· Evaluate the outcomes of translational research programs/mechanisms across the enterprise to identify and replicate the programs that are working.

· Fund access to core facilities and other resources and advertise these resources to the basic science community.

· Ensure that grant application review panels have the appropriate expertise to review translational research projects fairly. This may include creating separate translational research review groups or study sections and/or ensuring that translational researchers are among the scientists represented in review groups.

## Conclusion

Basic science is the foundation of medical advancement. Investigators with a deep understanding of fundamental biology and the mechanisms of disease are essential for translating laboratory discoveries into new and improved health interventions, diagnostics, and treatments. Additional training, resources, and support would enable basic scientists to move their discoveries forward effectively and efficiently. Although significant strides have been made, more can be done to optimize basic scientists’ participation in translational research. The research community should expand translational research training opportunities for basic researchers and trainees; facilitate their access to the funding, equipment, infrastructure, and other resources needed for translation; encourage and support collaboration between basic and clinical investigators across research disciplines and sectors; and recognize and reward basic scientists for the contributions that they make to this growing field. Implementing these recommendations will require action by research institutions and funders, scientific publishers, professional societies, and investigators themselves. Scientific societies such as FASEB and the many disciplinary societies representing biomedical researchers have a special role to play in advocating for these changes. We hope that this report will serve as a starting point for moving forward on this important issue.

## Endnotes

^a^National Institutes of Health. 2009. “NIH Announces New Program to Develop Therapeutics for Rare and Neglected Diseases.” Accessed May 30, 2011, at http://www.nih.gov/news/health/may2009/nhgri-20.htm.

## Competing interests

The symposium and the development and publication of this manuscript were sponsored by the Burroughs Wellcome Fund, the U.S. Department of Veterans Affairs, the Doris Duke Charitable Foundation, the Howard Hughes Medical Institute, and Merck Sharp & Dohme Corp.

## Authors’ contributions

JH and AD organized the meeting and prepared the manuscript. KH contributed to the preparation of the manuscript. RB, SC, BG, MM, SP UR, PR, JS, NS, PS, and RG served on the FASEB Translational Research Steering committee charged with overseeing the organization of the meeting and reviewing the manuscript, and RG was the chair of the steering committee. All authors read and approved the final manuscript.

## Supplementary Material

Additional file 1 Translational Research at NIH. Click here for file

Additional file 2 **FASEB Translational Research Symposium—Engaging Basic Scientists In Translational Research: Identifying Opportunities, Overcoming Obstacles.** Symposium agenda. Click here for file

Additional file 3 List of FASEB translational research symposium participants. Click here for file

Additional file 4 Highlights of a FASEB survey on participation in translational research. Click here for file

Additional file 5 Resources list that provides examples of policies, programs, and practices that could facilitate the engagement of basic scientists in translational research. Click here for file
