# Supplementary material for: Engaging basic scientists in translational research: identifying opportunities, overcoming obstacles
Source: J Transl Med. 2012 Apr 13;10:72. doi: 10.1186/1479-5876-10-72 (PMC3419626; doi:10.1186/1479-5876-10-72)
Supplement: Additional file 1 — Translational Research at NIH. [file 1479-5876-10-72-S1.pdf]

Translational Research at NIH

NIH Director Francis Collins, MD, PhD, opened the symposium with a presentation titled "Catalyzing Opportunities in Translational Research." He emphasized NIH's continued support for basic science and the important contributions it makes to the translational research pipeline. Dr. Collins also provided an overview of NIH efforts to promote and facilitate the movement of basic science discoveries into therapeutic applications. Those efforts are described below.

The **Therapeutics for Rare and Neglected Diseases** program encourages and speeds the development of new drugs for rare and neglected diseases. This unique program creates a drug development pipeline within NIH and is specifically intended to stimulate research collaborations with academic scientists, nonprofit organizations, and pharmaceutical and biotechnology companies working on rare and neglected illnesses.

The **Bridging Interventional Development Gaps** program (formerly the Rapid Access to Interventional Development [RAID] program) makes available, on a competitive basis, certain critical resources needed for the development of new therapeutic agents. Available services include the production, bulk supply, GMP manufacturing, formulation, and development of an assay suitable for pharmacokinetic testing and animal toxicology. Assistance also is provided with the regulatory process.

The **NIH Clinical Center** is the nation's largest hospital devoted entirely to clinical research. It conducts and supports research into the pathogenesis of disease; first-in-human clinical trials; the development of state-of-the-art diagnostic, preventive, and therapeutic interventions; and clinical research training.

The **Clinical and Translational Science Award (CTSA)** program creates a definable home for clinical and translational research at academic health centers across the nation. CTSA consortium institutions are building capabilities in connecting basic scientists with clinically experienced researchers to support training and to build a fully functional national network.

The **NIH-Food and Drug Administration Partnership** spearheads collaborative work on important public health issues. The Joint Leadership Council works together to help ensure that regulatory considerations form an integral component of biomedical research planning and that the latest science is integrated into the regulatory review process.

The **Cures Acceleration Network** was established to advance the development of high-need cures and reduce barriers to translation by providing new funding mechanisms and flexibility to help NIH more effectively manage projects.

The **National Center for Advancing Translational Sciences** brings together many of the above listed components, among others, into one cohesive entity. It will support partnerships to provide resources for investigators at all stages along the pipeline and enhance training and disciplines related to translational sciences, such as clinical pharmacology. The new Center is not intended to duplicate current activities in the other NIH Institutes and Centers, but rather to facilitate them and catalyze collaborations across the NIH enterprise.
