# Supplementary material for: Engaging basic scientists in translational research: identifying opportunities, overcoming obstacles
Source: J Transl Med. 2012 Apr 13;10:72. doi: 10.1186/1479-5876-10-72 (PMC3419626; doi:10.1186/1479-5876-10-72)
Supplement: Additional file 2 — FASEB Translational Research Symposium—Engaging Basic Scientists In Translational Research: Identifying Opportunities, Overcoming Obstacles. Symposium agenda. [file 1479-5876-10-72-S2.pdf]

FASEB Translational Research Symposium

Engaging Basic Scientists in Translational Research: Identifying Opportunities, Overcoming Obstacles

March 24-25, 2011

<u>March 24, 2011</u>

1:00-3:00 pm	**Registration**	Great Hall
3:00-3:30	**Welcome and meeting goals**<br>***Sean B. Carroll, PhD,*** *Vice President for Science Education, Howard Hughes Medical Institute*<br><br>***Richard Galbraith, MD, PhD,*** *Chair, FASEB Translational Research Steering Committee, Associate Dean, Patient Oriented Research, The University of Vermont College of Medicine*	Auditorium
3:30-4:30	**Remarks from the NIH Director**<br>***Francis Collins, MD, PhD,*** *Director, National Institutes of Health*	Auditorium
4:30-6:30	**What are the benefits of engaging basic scientists in translational research?**	Auditorium

Moderator: ***Paul Dechow, PhD,*** *Professor and Vice-Chair, Department of Biomedical Sciences, Baylor College of Dentistry, Texas A&M Health Science Center*

Team 1:
Cindy Farach-Carson, PhD, *Associate Vice Provost for Research, Rice University*
Daniel Wagner, PhD, *Assistant Professor, Department of Biochemistry and Cell Biology, Rice University*

Team 2:
Barry Coller, MD, *Vice President for Medical Affairs and Physician-in Chief, The Rockefeller University*
F. Nina Papavasiliou, PhD, *Associate Professor and Head of the Laboratory of Lymphocyte Biology, The Rockefeller University*
Thomas Tuschl, PhD, *Associate Professor and Head of the Laboratory for RNA Molecular Biology, The Rockefeller University*
Leslie Vosshall, PhD, *Robin Chemers Neustein Professor and Head of the Laboratory of Neurogenetics and Behavior, The Rockefeller University*

Team 3:
David Skorton, MD, *President, Cornell University*
Dirk Schnappinger, PhD, *Associate Professor of Microbiology and Immunology, Weill Cornell Medical College*

6:30-7:00	**Reception**	Great Hall
7:00-8:00	**Dinner and remarks by sponsors**<br>***Michael Rosenblatt, MD,*** *Executive Vice President and Chief Medical Officer, Merck & Co., Inc.*<br><br>***Betsy Myers, PhD,*** *Director, Medical Research Program, Doris Duke Charitable Foundation*<br><br>***Nancy S. Sung, PhD,*** *Senior Program Officer, Burroughs Wellcome Fund*<br><br>***Joel Kupersmith, MD,*** *Chief Research and Development Officer, Veterans Health Administration*	Dining Room
8:00-9:00	**Keynote presentation**<br>***Mary J.C. Hendrix, PhD,*** *Medical Research Institute Council Professor, President and Scientific Director, Children's Memorial Research Center, Robert H. Lurie Comprehensive Cancer Center, Northwestern University Feinberg School of Medicine*	Auditorium
9:00-9:05	**Closing remarks**<br>***Richard Galbraith, MD, PhD***	Auditorium
9:05-11:00	**Networking social**	Rathskeller

<u>March 25, 2011</u>

7:30-8:15 am	**Breakfast**	Dining Room
8:15-9:15	**Table discussions: Sparking interest in translational research**	Dining Room
9:15-11:30	**What are the challenges to engaging basic scientists in translational research?**	Auditorium

Survey results
John Smith, MD, PhD, MMM, DSc (Hon), *Professor of Pathology and Director, Division of Laboratory Medicine, University of Alabama at Birmingham*

Perspectives across career levels

Moderator: ***John Smith, MD, PhD, MMM, DSc (Hon)***

Panelists:

Michael Dyer, PhD, *Co-Leader, Developmental Therapeutics for Solid Malignancies Program, St. Jude Children's Research Hospital*

Daria Mochly-Rosen, PhD, *Professor and Senior Associate Dean for Research and Director of SPARK, Stanford's Translational Research Program, Stanford University, School of Medicine*

Philip Haydon, PhD, *Annetta and Gustav Grisard Professor and Chair, Department of Neuroscience, Tufts University School of Medicine*

Robert J. Alpern, MD, *Ensign Professor of Medicine and Dean, Yale School of Medicine*

11:30-12:15 pm	**Lunch**		Dining Room
12:15-2:00	**Breakout sessions: How can the research community address these challenges?**		
	**Breakout 1:**	**Facilitating translational research training**	Auditorium
	**Moderator:**	***William Galey, PhD,*** *Director, Graduate and Medical Education Programs, Howard Hughes Medical Institute*	
	Panelists:	***Khandan Keyomarsi, PhD,*** *Professor, Department of Experimental Radiation Oncology, The University of Texas MD Anderson Cancer Center (Initiative: TRIUMPH Postdoctoral Fellowship)*	
		Matthew J. Walter, MD, *Assistant Professor of Medicine and Genetics, Washington University School of Medicine (Initiative: Lucille P. Markey Special Emphasis Pathway in Human Pathobiology)*	
		Mary Estes, PhD, *Professor, Department of Molecular Virology and Microbiology, Baylor College of Medicine (Initiative: HHMI Med into Grad)*	
		Rebecca Jackson, MD, *Professor and Associate Dean, College of Medicine, The Ohio State University (Initiative: CTSA Consortium Training and Career Development of Clinical/Translational Scientists)*	
	**Breakout 2:**	**Facilitating productive collaborations**	Small Auditorium (K202)
	**Moderator:**	***Richard Bockman, MD, PhD,*** *Professor of Medicine, Endocrine Division, Weill Cornell Medical College, and Chief of the Endocrine Service, Hospital for Special Surgery*	
	Panelists:	***Gail Cassell, PhD,*** *Visiting Professor, Department of Global Health and Social Medicine, Harvard Medical School and Vice President of TB Drug Discovery, Infectious Disease Research Institute in Seattle*	
		Scott J. Weir, PharmD, PhD, *Director, Institute for Advancing Medical Innovation, University of Kansas Cancer Center*	
		Lee M. Nadler, MD, *Virginia and D.K. Ludwig Professor of Medicine, Harvard Medical School*	
		Jeffrey Bluestone, PhD, *A.W. and Mary Margaret Clausen Distinguished Professor, Executive Vice Chancellor, and Provost, University of California, San Francisco*	
	**Breakout 3:**	**Providing appropriate recognition and rewards**	D124
	**Moderator:**	***Patrick Stover, PhD,*** *Professor and Director, Division of Nutritional Sciences, Cornell University*	
	Panelists:	***Sally Kornbluth, PhD,*** *Vice Dean for Research, James B. Duke Professor, Pharmacology and Cancer Biology, Duke University*	

School of Medicine

Francesco Marincola, MD, *Editor-in-Chief, Journal of Translational Medicine and Chief, Infectious Disease and Immunogenetics Section, Department of Transfusion Medicine, National Institutes of Health Clinical Center*

Paul DiCorleto, PhD, *The Sherwin-Page Chair, Cleveland Clinic Lerner Research Institute*

Breakout 4: The role of funding organizations — D125

Moderator: ***Nancy S. Sung, PhD,*** *Senior Program Officer, Burroughs Wellcome Fund*

Panelists: ***Barbara Alving, MD,*** *Director, National Center for Research Resources, National Institutes of Health*

Sally McNagny, MD, MPH, FACP, *Vice President, The Medical Foundation, a division of HRiA*

Joel Kupersmith, MD, *Chief Research and Development Officer, Veterans Health Administration*

Dennis McKearin, PhD, *Senior Scientific Officer, Howard Hughes Medical Institute*

2:00-2:15	**Break**	Great Hall
2:15-3:00	**Recommendations from breakout groups**<br>***Breakout session moderators***	Auditorium
3:00-3:15	**Wrap up and next steps**<br>***Richard Galbraith, MD, PhD***	Auditorium
