# Supplementary material for: Engaging basic scientists in translational research: identifying opportunities, overcoming obstacles
Source: J Transl Med. 2012 Apr 13;10:72. doi: 10.1186/1479-5876-10-72 (PMC3419626; doi:10.1186/1479-5876-10-72)
Supplement: Additional file 3 — List of FASEB translational research symposium participants. [file 1479-5876-10-72-S3.pdf]

FASEB Translational Research Symposium
Engaging Basic Scientists in Translational Research: Identifying Opportunities, Overcoming Obstacles

Meeting Participants

Myles Akabas, MD, PhD
Professor of Physiology & Biophysics
Director, Medical Scientist Training Program
Albert Einstein College of Medicine of Yeshiva University
myles.akabas@einstein.yu.edu

Robert Alpern, MD
Dean
Yale School of Medicine
robert.alpern@yale.edu

Bruce Altevogt, PhD
Senior Program Officer
Institute of Medicine
baltevogt@nas.edu

Barbara Alving, MD, MACP
Director
National Center for Research Resources
National Institutes of Health
alvingb@mail.nih.gov

Margaret Anderson, MS
Executive Director
FasterCures
manderson@fastercures.org

James C. Austin, PhD
Editor, *Science Careers*
AAAS
jaustin@aaas.org

Robert Berdahl, PhD
President
Association of American Universities
robert_berdahl@aau.edu

Daniel Bernard, PhD
Associate Professor
McGill University
daniel.bernard@mcgill.ca

Jim Bernstein, MPA
Director
Government & Public Affairs
American Society for Pharmacology & Experimental Therapeutics
Jbernstein@aspet.org

Sanjay Bidichandani, MBBS, PhD
Vice President for Research
Muscular Dystrophy Association
sbidichandani@mdausa.org

Linda Birnbaum, PhD
Director
National Institute of Environmental Health Sciences
National Institutes of Health
birnbaumls@niehs.nih.gov

Mitsi Blount, PhD
Assistant Professor
Emory University
mabloun@emory.edu

Jeffrey Bluestone, PhD
Professor, Executive Vice Chancellor, and Provost
University of California San Francisco
jeff.bluestone@ucsf.edu

Richard Bockman, MD, PhD
Professor of Medicine
Weill Medical College/Hospital for Special Surgery
bockmanr@hss.edu

Jo Boughman, PhD
Executive Vice President
American Society of Human Genetics
jboughman@ashg.org

Pamela Bradley, PhD
Associate Director of Science Policy
American Association for Cancer Research
pamela.bradley@aacr.org

Linda Brady, PhD
Director, Division of Neuroscience and Basic Behavioral Science
National Institute of Mental Health
lbrady@mail.nih.gov

Brian Brewer
Director of Communications
Cancer Research Institute
bbrewer@cancerresearch.org

PJ Brooks, PhD
Program Officer and Adjunct Investigator
National Institute on Alcohol Abuse and Alcoholism and Office of Rare Diseases Research
National Institutes of Health
pjbrooks@mail.nih.gov

Scott Campbell, PhD
Executive Director and CEO
Foundation for the National Institutes of Health
scampbell@fnih.org

Maria Teresa Canto, DDS, MS, MPH
Health Scientist Administrator
National Center for Research Resources
National Institutes of Health
cantom@mail.nih.gov

Sean Carroll, PhD
Vice President for Science Education
Howard Hughes Medical Institute
carrolls@hhmi.org

Robert Carter, MD, PhD
Deputy Director
National Institute of Arthritis and Musculoskeletal and Skin Diseases
National Institutes of Health
carterrob@mail.nih.gov

Gail Cassell, PhD
Visiting Professor
Department of Global Health and Social Medicine Harvard Medical School and
Vice President of TB Drug Discovery
Infectious Disease Research Institute in Seattle
gail.h.cassell@gmail.com

John Chatham, PhD
Professor and Director
Molecular and Cellular Pathology
University of Alabama at Birmingham
jchatham@uab.edu

Clement Chow, PhD
Postdoctoral Scientist
Cornell University
cyc8@cornell.edu

Ida Chow, PhD
Executive Officer
Society for Developmental Biology
ichow@sdbonline.org

Stanley Cohen, MD
Chair
Department of Pathology
UMDNJ-New Jersey Medical School
cohenst@umdnj.edu

Barry Coller, MD
Physician-in-Chief and Vice President for Medical Affairs
The Rockefeller University
collerb@rockefeller.edu

Francis Collins, MD, PhD
Director
National Institutes of Health
collinsf@mail.nih.gov

Angela Colmone, PhD
Associate Editor
Science Translational Medicine
acolmone@aaas.org

Leslie Cooper, RN, BSN, MPH, PhD
Program Officer
National Center for Research Resources
National Institutes of Health
lc58q@nih.gov

Milton Corn, MD
Deputy Director for Research and Education
National Library of Medicine
National Institutes of Health
cornm@mail.nih.gov

Kyle Covington
Graduate Student
Baylor College of Medicine
covingto@bcm.edu

James M. Crawford, MD, PhD
Senior Vice President
Pathology & Laboratory Medicine
North Shore-Long Island Jewish Health System
Laboratories
bdeclem@nshs.edu

Meredith Crosby, PhD
Postdoctoral Scientist
Yale University/Procter & Gamble
meredithcrosby@hotmail.com

Geraldine Dawson, PhD
Chief Science Officer
Autism Speaks
elizabeth.sturdivant@autismspeaks.org

Paul Dechow, PhD
Professor and Vice-Chair
Department of Biomedical Sciences
Texas A&M Health Science Center
Baylor College of Dentistry
pdechow@bcd.tamhsc.edu

Anne M. Deschamps, PhD
Science Policy Analyst
Federation of American Societies for Experimental Biology
adeschamps@faseb.org

Paul DiCorleto, PhD
Chair, Lerner Research Institute
Cleveland Clinic
dicorlp@ccf.org

Loretta Doan, PhD
Director, Science Policy
The Endocrine Society
ldoan@endo-society.org

Chhanda Dutta, PhD
Chief, Clinical Gerontology Branch
Division of Geriatrics and Clinical Gerontology
National Institute on Aging
National Institutes of Health
duttac@mail.nih.gov

Steven Dworkin, PhD
Professor and Chair of Psychology
Western Illinois University
si-dworkin@wiu.edu

Michael Dyer, PhD
Member
St. Jude Children's Research Hospital
michael.dyer@stjude.org

David J. Eckstein, PhD
Senior Health Scientist Administrator
Office of Rare Diseases Research
National Institutes of Health
eckstein@mail.nih.gov

Joel Ernst, MD
Professor
NYU School of Medicine
joel.ernst@med.nyu.edu

Sindy Escobar-Alvarez, PhD
Program Officer for Medical Research
Doris Duke Charitable Foundation
sescobar@ddcf.org

Mary Estes, PhD
Professor of Molecular Virology and Co-Director
Graduate Program in Translational Biology and Molecular Medicine
Baylor College of Medicine
mestes@bcm.edu

Mary C. Farach-Carson, PhD
Associate Vice Provost for Research
Rice University
farachca@rice.edu

Fred Finkelman, MD
McDonald Professor of Medicine
Professor of Pediatrics
University of Cincinnati College of Medicine
ffinkelman@pol.net

Michael Fleming, MD, MPH
Professor
Northwestern University
m-fleming@northwestern.edu

Susan Foy
Graduate Student
Cleveland Clinic Lerner College of Medicine of Case Western Reserve University
westers@ccf.org

Martin Frank, PhD
Executive Director
American Physiological Society
mfrank@the-aps.org

Maryrose Franko, PhD
Senior Program Officer
Howard Hughes Medical Institute
frankom@hhmi.org

Richard Galbraith, MD, PhD
Professor of Medicine and Associate Dean
University of Vermont
richard.galbraith@uvm.edu

Bill Galey, PhD
Director of Graduate and Medical Education Programs
Howard Hughes Medical Institute
galeyw@hhim.org

Nao Gamo, MPhil
Graduate Student
Yale University
nao.gamo@yale.edu

Howard Garrison, PhD
Director, Office of Public Affairs
Federation of American Societies for Experimental Biology
hgarrison@faseb.org

Eric Green, MD, PhD
Director
National Human Genome Research Institute
National Institutes of Health
egreen@nhgri.nih.gov

Lawrence Green
Communications Specialist
Federation of American Societies for Experimental Biology
lgreen@faseb.org

Nadja Grobe, PhD
Postdoctoral Research Fellow
Wright State University
nadja.grobe@wright.edu

Lauren Gross, JD
Director of Public Policy and Government Affairs
The American Association of Immunologists
lgross@aai.org

Lisa Guay-Woodford, MD
Professor and Vice Chair
University of Alabama at Birmingham
lgw@uab.edu

Kathi Hanna, PhD
Science and Health Policy Consultant
hanna.kathi@gmail.com

Rebecca Hartley, PhD
Associate Professor
University of New Mexico Health Sciences Center
rhartley@salud.unm.edu

Philip Haydon, PhD
Annetta and Gustav Grisard Professor and Chair
Department of Neuroscience
Tufts University School of Medicine
philip.haydon@tufts.edu

Anthony Hayward, MD, PhD
Director, Division for Clinical Research Resources
National Center for Research Resources
National Institutes of Health
haywarda@mail.nih.gov

Joseph (JR) Haywood, PhD
Chair, Deparment of Pharmacology
Michigan State University
haywoo12@msu.edu

Terry Hébert, PhD
Associate Professor
McGill University
terence.hebert@mcgill.ca

Mary J.C. Hendrix, PhD
President & Scientific Director
Children's Memorial Research Center
m-hendrix@northwestern.edu

Jennifer A. Hobin, PhD
Director of Science Policy
Federation of American Societies for Experimental Biology
jhobin@gmail.com

Marybeth Howlett, MEM
Managing Director
The Society of Nuclear Medicine
mhowlett@snm.org

Valerie Hu, PhD
Professor of Biochemistry and Molecular Biology
The George Washington University Medical Center
bcmvwh@gwumc.edu

Geoff Hunt, PhD
Science Policy Fellow
American Society for Biochemistry & Molecular Biology
ghunt@asbmb.org

Richard Insel, MD
Chief Scientific Officer
Juvenile Diabetes Research Foundation
rinsel@jdrf.org

Rebecca Jackson, MD
Associate Dean for Clinical Research
The Ohio State University
Rebecca.Jackson@osumc.edu

Joseph Kemnitz, PhD
Director, Translational Technologies and Resources
University of Wisconsin Madison
jkemnitz@wisc.edu

Khandan Keyomarsi, PhD
Professor
University of Texas MD Anderson Cancer Center
kkeyomar@mdanderson.org

Robert Kimberly, MD
Senior Associate Dean for Research
University of Alabama at Birmingham
rpk@uab.edu

Stephen Korn, PhD
Director of Training and Career Development
National Institute of Neurological Disorders and Stroke
National Institutes of Health
korns@ninds.nih.gov

Sally Kornbluth, PhD
Vice Dean for Research
Duke University School of Medicine
kornb001@mc.duke.edu

Henry Krause, PhD
Professor
University of Toronto
h.krause@utoronto.ca

Joel Kupersmith, MD
Chief Research & Development Officer
U.S. Department of Veterans Affairs
nazneen.mama@yahoo.com

Michael Kurilla, MD, PhD
Director
National Institute of Allergy and Infectious Diseases
National Institutes of Health
mkurilla@niaid.nih.gov

Jennifer Kwan
Policy Chair
American Physician Scientists Association
M.D.-Ph.D. Candidate
Medical Scientist Training Program
University of Illinois at Chicago
kwanjen@gmail.com

Megan Lassig
Program Assistant
Howard Hughes Medical Institute
lassigm@hhmi.org

Anh-Chi Le, PhD
Program Officer
Howard Hughes Medical Institute
leanh@hhmi.org

Stephanie Lederman, EdM
Executive Director
American Federation for Aging Research
stephanie@afar.org

Grace Lee, PhD
Pharmacology/Toxicology Reviewer
U.S. Food and Drug Administration
grace.lee@fda.hhs.gov

Yung Lie, PhD
Scientific Director
Damon Runyon Cancer Research Foundation
yung.lie@damonrunyon.org

Yvonne Maddox, PhD
Deputy Director
National Institute of Child Health and Human Development
National Institutes of Health
maddoxy@mail.nih.gov

Marc Malandro, PhD
Associate Vice Chancellor for Technology Management and Commercialization
University of Pittsburgh
msm31@pitt.edu

Francesco Marincola, MD
Tenured Senior Investigator
Clinical Center Department of Transfusion Medicine
National Institutes of Health
fmarincola@mail.cc.nih.gov

Priscilla Markwood, CAE
Director of Scientific Affairs, Communications & Society Services
American Society for Investigative Pathology
pmarkwood@asip.org

Bettie Sue Masters, PhD
The Robert A. Welch Distinguished Chair in Chemistry
University of Texas Health Science Center San Antonio
masters@uthscsa.edu

Donald McClain, MD, PhD
Program Director
University of Utah
donald.mcclain@hsc.utah.edu

Dennis McKearin, PhD
Senior Scientific Officer
Howard Hughes Medical Institute
mckearind@hhmi.org

Matthew McMahon, PhD
Senior Advisor for Translational Research
National Eye Institute
National Institutes of Health
mm@nei.nih.gov

Sally McNagny, MD, MPH
Vice President
The Medical Foundation, a division of HRiA
smcnagny@hria.org

Elizabeth McNally, MD, PhD
Professor of Medicine and Human Genetics
Director, Institute for Cardiovascular Research
Director, Cardiovascular Genetics Clinic
President Elect
American Society for Clinical Investigation
emcnally@uchicago.edu

Francis Miller, MD
Associate Professor of Medicine
University of Iowa
francis-miller@uiowa.edu

Thomas Miller, PhD
Program Director
National Institute of Neurological Disorders and Stroke
National Institutes of Health
tm208y@nih.gov

Daria Mochly-Rosen, PhD
Senior Associate Dean for Research
Professor Chemical & Systems Biology
Stanford University
mochly@stanford.edu

Katie Moore, PhD
Manager, Science Policy
The Endocrine Society
kmoore@endo-society.org

Mariana Morris, PhD
Professor and Chair
Assistant Vice President for Graduate Studies
Wright State University
mariana.morris@wright.edu

Cynthia Morton, MD
Professor of Obstetrics/Gynecology and Pathology
Brigham and Women's Hospital
Harvard Medical School
cmorton@partners.org

Karen Mowrer, PhD
Legislative Affairs Officer
Federation of American Societies for Experimental Biology
kmowrer@faseb.org

James Musser, MD, PhD
Chair
Department of Pathology and Laboratory Medicine
The Methodist Hospital
JMMusser@tmhs.org

Elizabeth Myers, PhD
Director, Medical Research Program
Doris Duke Charitable Foundation
emyers@ddcf.org

Lee Nadler, MD
Dean of Clinical and Translational Research
Harvard Medical School
lee_nadler@dfci.harvard.edu

Kim Ngo
Public Affairs Assistant
Federation of American Societies for Experimental Biology
kngo@faseb.org

Margaret Offermann, MD, PhD
Principal
The Salutramed Group
mkoffermann@gmail.com

Frederick Ognibene, MD
Deputy Director Educational Affairs & Strategic Partnerships
National Institutes of Health
fognibene@cc.nih.gov

Alex Ommaya, DSc
Director Translational Research
U.S. Department of Veterans Affairs
alex.ommaya@va.gov

Rebecca Osthus, PhD
Science Policy Analyst
American Physiological Society
rosthus@the-aps.org

F. Nina Papavasiliou, PhD
Associate Professor and Head of the Laboratory Of Lymphocyte Biology
The Rockefeller University
Nina.Papavasiliou@rockefeller.edu

Todd Peterson, PhD
Assistant Professor
Vanderbilt University
todd.e.peterson@vanderbilt.edu

Sharma Prabhakar, MD, MBA, FACP
Professor and Chief of Nephrology
Vice-Chair, Dept of Medicine
Texas Tech University Health Sciences Center
sharma.prabhakar@ttuhsc.edu

M. Renee Prater, PhD, DVM
Associate Professor and Chair of Immunology
Edward Via College of Osteopathic Medicine
mrprater@vt.edu

Alice Ra'anan
Director of Science Policy
American Physiological Society
araanan@the-aps.org

J. Usha Raj, MD
Professor and Head
University of Illinois at Chicago
usharaj@uic.edu

Marc Reitman, MD, PhD
Branch Chief
National Institute of Diabetes and Digestive and Kidney Diseases
National Institutes of Health
marc.reitman@nih.gov

Makeda Richardson
Program Assistant
Graduate Science Education
Howard Hughes Medical Institute
richardsonm@hhmi.org

Rose Marie Robertson, MD
Chief Science Officer
American Heart Association
rosemarie.robertson@heart.org

Michael Rosenblatt, MD
Executive Vice President
Chief Medical Officer
Merck & Co.
betty.malloy@merck.com

Theodora Ross, MD, PhD
Professor
University of Michigan
tsross@umich.edu

Doris Rubio, PhD
Professor of Medicine
University of Pittsburgh
rubiodm@upmc.edu

Alisa Schaefer, PhD
Advisor for Global Outreach
National Institutes of Health
schaefera@mail.nih.gov

Dirk Schnappinger, PhD
Associate Professor of Microbiology and Immunology
Weill Cornell Medical College
dis2003@med.cornell.edu

Jacob Schumacher
Legislative Aide
The American Association of Immunologists
jschumacher@aai.org

Harry Shamoon, MD
Associate Dean
Clinical and Translational Research
Albert Einstein College of Medicine
harry.shamoon@einstein.yu.edu

Douglas Sheeley, ScD
Senior Scientific Officer
National Center for Research Resources
National Institutes of Health
dms@nih.gov

Susan Shurin, MD
Acting Director
National Heart, Lung, and Blood Institute
National Institutes of Health
shurinsb@nhlbi.nih.gov

Hadley Sikes, PhD
Assistant Professor
Massachusetts Institute of Technology
sikes@mit.edu

David Skorton, MD
President
Cornell University
teri.burdick@cornell.edu

Jacqueline Slavik, PhD
Executive Director
Biomedical Research Institute
Brigham and Women's Hospital
jslavik@partners.org

Bonnie Sloane, PhD
Distinguished Professor & Chair
Wayne State University
bsloane@med.wayne.edu

John A. Smith, MD, PhD, MMM, DSC (Hon)
Director, Division of Laboratory Medicine
Professor of Pathology
University of Alabama at Birmingham
jas@uab.edu

Mark Sobel, MD, PhD
Executive Officer
American Society for Investigative Pathology
mesobel@asip.org

Anita Miller Sostek, PhD
Vice President for Scientific Review and Operations
Autism Speaks
anita.sostek@autismspeaks.org

Tyrone Spady, PhD
Science Policy Analyst
Federation of American Societies for Experimental Biology
tspady@faseb.org

Patrick Stover, PhD
Professor and Director
Division of Nutritional Sciences
Cornell University
pjs13@cornell.edu

Nancy Sung, PhD
Senior Program Officer
Burroughs Wellcome Fund
nsung@bwfund.org

Bernard Talbot, MD, PhD
Medical Officer
National Center for Research Resources
National Institutes of Health
talbotb@mail.nih.gov

William Talman, MD
Professor of Neurology and Neuroscience
University of Iowa
william-talman@uiowa.edu

Thomas Tuschl, PhD
Associate Professor and Head of the Laboratory for RNA Molecular Biology
The Rockefeller University
ttuschl@rockefeller.edu

Mark Tykocinski, MD
Dean
Jefferson Medical College
mark.tykocinski@jefferson.edu

Rod Ulane, PhD
NIH Research Training Officer
National Intitutes of Health
ulanere@od.nih.gov

Leslie Vosshall, PhD
Robin Chemers Neustein Professor and Head of the Laboratory of Neurogenetics and Behavior
The Rockefeller University
Leslie.Vosshall@rockefeller.edu

Daniel Wagenaar, PhD
Broad Senior Research Fellow in Brain Circuitry
California Institute of Technology
daw@caltech.edu

Daniel Wagner, PhD
Assistant Professor, Department of Biochemistry and Cell Biology
Rice University
dswagner@rice.edu

Matt Walter, MD
Assistant Professor of Medicine
Washington University School of Medicine
mjwalter@dom.wustl.edu

Ena Wang, MD
Staff Scientist
Clinical Center Department of Transfusion Medicine
National Institutes of Health
ewang@mail.nih.gov

Scott Weir, PharmD, PhD
Director
Institute for Advancing Medical Innovation University of Kansas
sweir@kumc.edu

Jennifer Westendorf, PhD
Associate Professor
Mayo Clinic
westendorf.jennifer@mayo.edu

Andrea Widener, MA
Science Writer
Howard Hughes Medical Institute
widenera@hhmi.org

Heng Xie, MD, MPH, PhD
Supervisory Medical Officer
National Center for Research Resources
National Institutes of Health
xiehe@mail.nih.gov

Wen Xie, MD, PhD
Associate Professor
University of Pittsburgh
wex6@pitt.edu

Liming Yang, PhD
Program Officer
National Center for Research Resources
National Institutes of Health
lyang@mail.nih.gov

Jennifer Zeitzer
Director of Legislative Relations
Federation of American Societies for Experimental Biology
jzeitzer@faseb.org
