# Supplementary material for: Engaging basic scientists in translational research: identifying opportunities, overcoming obstacles
Source: J Transl Med. 2012 Apr 13;10:72. doi: 10.1186/1479-5876-10-72 (PMC3419626; doi:10.1186/1479-5876-10-72)

FASEB Translational Research Survey

FASEB conducted a survey of a sample of its societies' members and meeting attendees to assess scientists' interest and participation in translational research, as well as the actual and perceived challenges of conducting translational science. The survey garnered 1,770 complete responses.

Who responded?

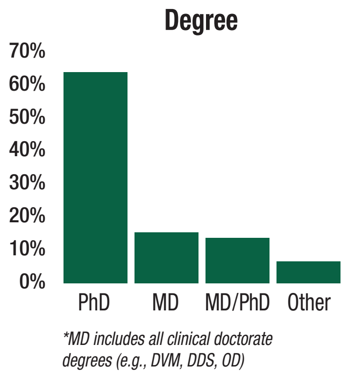

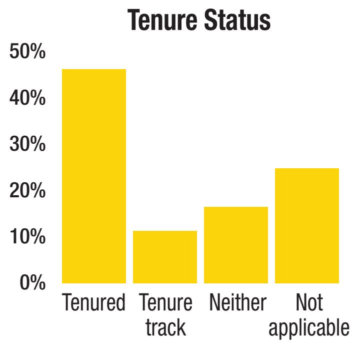

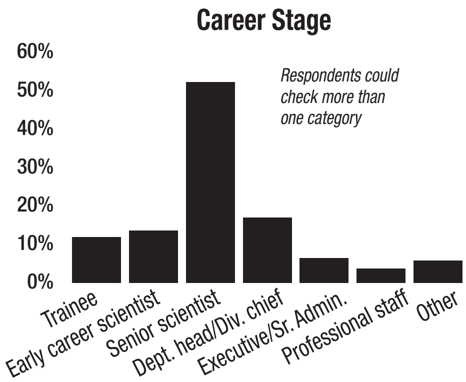

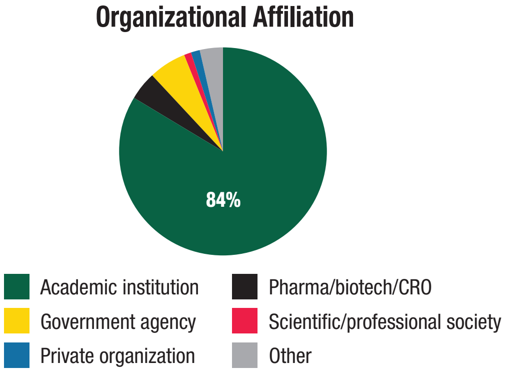

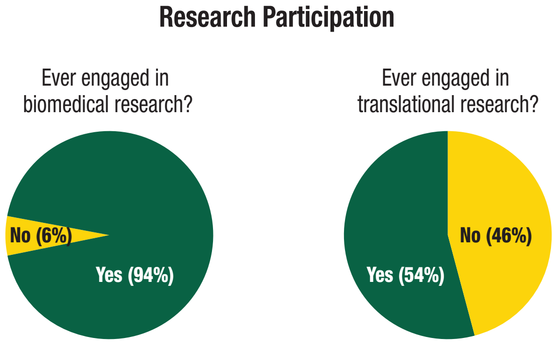

How did they describe their research programs?

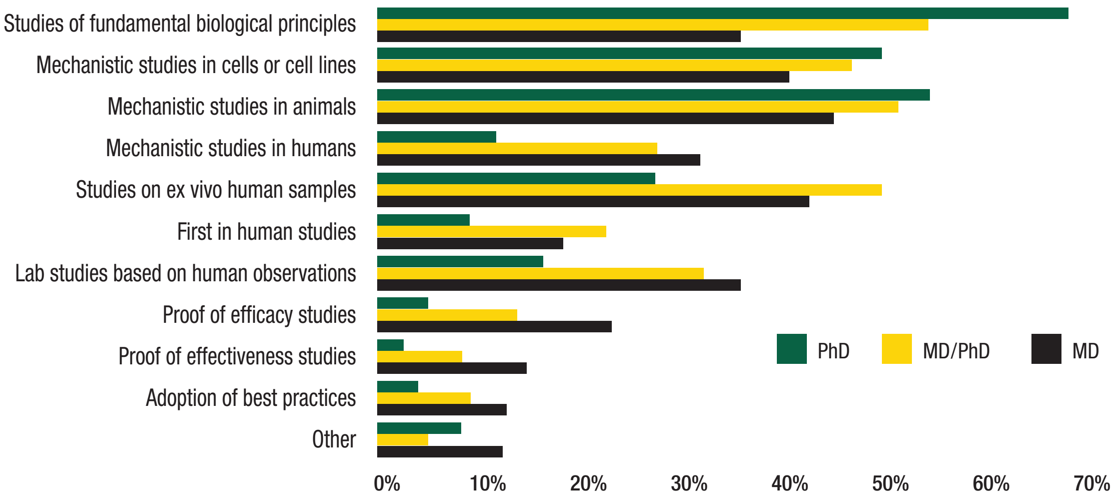

Why did they get involved in translational research?

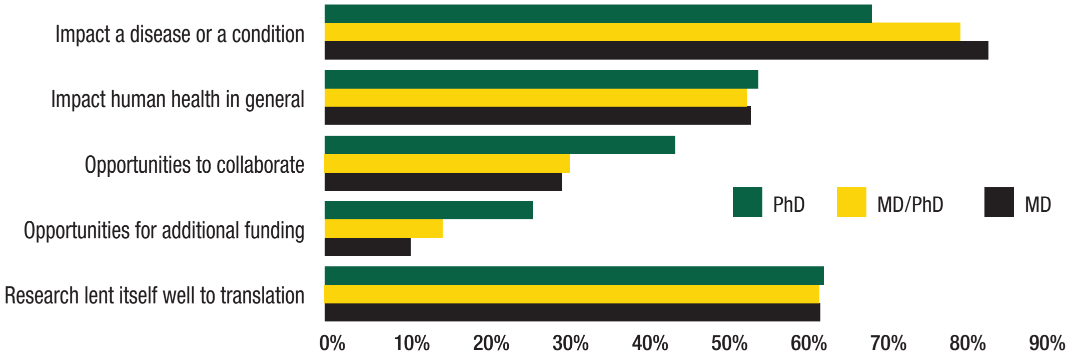

What challenges did they face?

Respondents were asked to rate how difficult it was to overcome barriers to conducting translational research on a scale of 1(not at all difficult) through 7 (extremely difficult). The top graph shows the ratings for all participants, whether they had conducted translational research (actual barriers, A) or had not conducted translational research (perceived barriers, P). The bottom graph shows only the challenges that were rated differently by the two groups based on Chi Square analysis, p<.05.

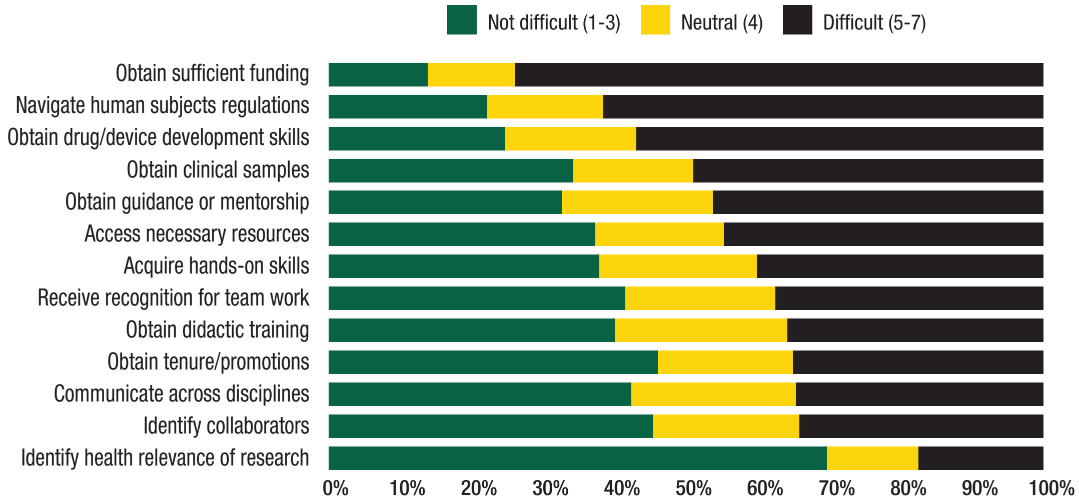

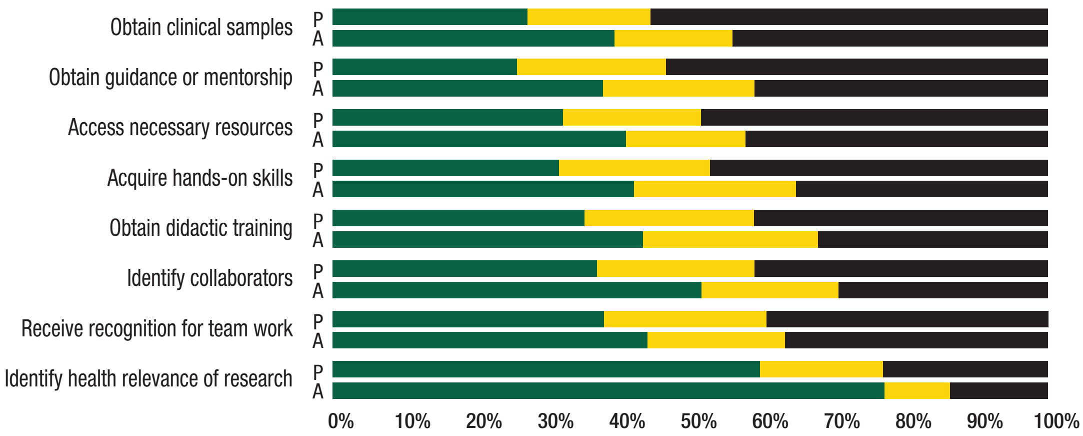

Supplement: Additional file 4 — Highlights of a FASEB survey on participation in translational research. [file 1479-5876-10-72-S4.pdf]
