# Supplementary material for: Engaging basic scientists in translational research: identifying opportunities, overcoming obstacles
Source: J Transl Med. 2012 Apr 13;10:72. doi: 10.1186/1479-5876-10-72 (PMC3419626; doi:10.1186/1479-5876-10-72)
Supplement: Additional file 5 — Resources list that provides examples of policies, programs, and practices that could facilitate the engagement of basic scientists in translational research. [file 1479-5876-10-72-S5.pdf]

Resources to Facilitate Participation in Translational Research

This appendix supplements the report recommendations by providing examples of policies, programs, and practices that could facilitate the engagement of basic scientists in translational research. It is not intended to be a comprehensive list. Rather, it aims to give a general "flavor" of the types of approaches institutions, including universities, funding organizations, and professional societies, may want to adopt.

**Establishing Collaborations**			
**Resource, Policy, or Program**	**Organization (weblink)**	**Description**	**Purpose**
**BiomedExperts**	BiomedExperts<br>http://www.biomedexperts.com	Enables social networking to help scientists share data and search for potential collaborators based on their areas of expertise and professional networks.	Identify collaborators and expertise; share data
**Collaboration and Team Science: A Field Guide**	National Institutes of Health<br>http://tinyurl.com/75r8foz	Provides assistance to investigators who are participating on, leading, or contemplating building a research team. Topics include building a research team, developing a shared vision, sharing recognition and credit, and handling conflict.	Provide information on collaboration and team science
**Collaborative Agreement Template**	National Institutes of Health<br>http://tinyurl.com/cdohgwm	Provides a template for developing a collaborative agreement that explicitly states the team's expectations, goals, and values. This template is contained in NIH's "Collaboration and Team Science: A Field Guide" (see above).	Facilitate collaborative agreements
**Global Centers for Therapeutic Innovation**	Pfizer,<br>University of California San Francisco<br>http://tinyurl.com/4mlmdhp	Network of partnerships with academic medical centers (AMCs) designed to develop biologic therapeutic candidates. Centers are jointly staffed by Pfizer employees and basic and translational scientists and doctoral candidates from AMCs. Pfizer provides investigators with access to its resources and incentivizes participation through milestone payments, publishing rights, and intellectual property use.	Foster collaboration to promote drug development

**Harvard Catalyst Profiles**	Harvard University http://tinyurl.com/7lkqyaj	Provides a research networking and expertise mining software tool that connects people by combining basic directory information with expertise keywords. Each person in the network has a profile page that includes his or her name, titles, affiliations, and contact information. Enables the creation of automatically formed passive networks based on shared traits (e.g., publications, departments) and active networks defined by the user.	Identify expertise, collaborators
**Research "Speed Dating"**	Multiple, including Cornell and Dalhousie universities http://tinyurl.com/7h3b82o; http://tinyurl.com/7fouycf	Describes speed networking sessions that aim to spark collaborations across disciplines and institutions. Participants (e.g., academic investigators, industry scientists, physicians) are given two to three minutes to exchange cards, biographies, and research interests before moving on to share the same information with the next participant.	Identify expertise, collaborators
**Research Team Support and Development**	Northwestern University Clinical and Translational Sciences Institute http://tinyurl.com/6rxxsqh	Offers collaboration enhancement for team-based, cross-disciplinary translational biomedical research and helps translate research findings about team science into effective practices for teams and funders. Resources include expert/collaborator identification and referral, collaboration facilitation, team science tools and resources, collaborative research funding information, collaborative research program development, and team science training.	Provides support for team science
**SciVal Experts**	SciVal Experts http://preview.tinyurl.com/7pn9pd2	Provides a directory of research expertise prepopulated with publication histories from Elsevier's Scopus database. It provides a visual analysis of relevant concepts that instantly exposes a researcher's distinctive expertise. It also can incorporate institutional data and individual researcher updates.	Identify expertise, collaborators
**VIVO**	Multiple; available to institutions with relevant technology http://www.vivoweb.org	Provides an open source semantic web application that enables the discovery of research and scholarship across disciplines and institutions by creating a semantic cloud of information that can be searched and browsed. It is populated with detailed profiles of faculty and researchers including information on publications, teaching, service, and professional affiliations.	Identify research, scholarship, and collaborators

Identifying Research Tools and Resources			
**Resource, Policy, or Program**	**Organization (weblink)**	**Description**	**Purpose**
**CTSA Pharmaceutical Assets Portal**	Multiple http://www.ctsapharmaportal.org/aboutus.html	Facilitates matches between CTSA-affiliated investigators and the pharmaceutical industry to develop research partnerships based on potentially valuable compounds that did not make it to the market. Academic researchers can submit proposals to pharma partners requesting a specific drugs or a drug category with a desired effect. Pharma partners can contact target experts identified from clusters of researchers organized around areas of expertise.	Identify research compounds, expertise, and collaborators
**eagle-i**	Multiple; consortium of nine member institutions https://www.eagle-i.net/	Prototype of a national research resource discovery network to help biomedical scientists search for and find previously unknown, but highly valuable, resources. Resources accessible through the site include animal models, reagents, cell and tissue banks, core facilities, and training opportunities.	Identify research resources
**SPARK**	Stanford University http://sparkmed.stanford.edu	Provides funding, infrastructure, and expertise to move research with therapeutic applications toward the clinic. SPARK fellows receive education and training; guidance on protocol development, trial design, intellectual property law, consent form design, and regulatory document submission from professionals in academe and industry; and assistance accessing core facilities.	Provide funding, infrastructure, expertise, and training
**Translational Research Training**			
**Resource, Policy, or Program**	**Organization (weblink)**	**Description**	**Purpose**
**COALESCE**	Northwestern University Clinical and Translational Science Center http://teamscience.net	Aims to create, evaluate, and disseminate online learning resources to enhance skills needed to perform transdisciplinary, team-based translational research. Learning modules help users acquire and apply knowledge of team science and provide an experiential learning environment where they can engage virtually in the challenges of team research.	Educate and train researchers and institutional development officers in team research

**Clinical and Translational Research Summer Seminar Series**	Northwestern University http://tinyurl.com/bpwxaag	Provides a two-day program to educate medical students and undergraduates in the skills required for biomedical research. It covers topics including, biostatistics, epidemiology, responsible conduct of research, human subjects research, the research enterprise, clinical trial conduct, drug and device development, and career opportunities.	Educate undergraduate and medical students
**Core Competencies for Clinical and Translational Research**	CTSA Consortium http://tinyurl.com/cyoxukh	A set of competencies within 14 thematic areas that define the skills, attributes, and knowledge needed to conduct clinical and translational research. The competencies can serve as a guide for anyone considering developing a translational curriculum at multiple graduate levels.	Educate graduate students, postdoctoral researchers, and independent investigators
**Demystifying Medicine**	National Institutes of Health http://demystifyingmedicine.od.nih.gov/	A weekly, one-hour course for PhDs that includes presentation of patients, pathology, diagnosis, and therapy in the context of major disease problems and current research. The course is designed to help bridge the gap between advances in biology and their application to major human diseases. Each session includes clinical and basic science components presented by staff and outside invitees.	Educate graduate students, postdoctoral researchers, and independent investigators
**Lucille P. Markey Special Emphasis Pathway in Human Pathobiology Program**	Washington University in St. Louis http://dbbs.wustl.edu/markeypathway	Two-year course of study for graduate students and postdoctorates that supplements biomedical PhD education. It includes a course in Pathobiology of Human Disease States, individualized clinical mentorship, and an annual retreat enabling personal and scientific interactions between trainees and faculty.	Educate and train PhD students and postdoctoral researchers

**Masters of Science in Medicine Degree Program**	Stanford University http://msm.stanford.edu/	Provides PhD students with exposure to medicine to foster translational research. Students are required to complete PhD course requirements and lab rotations. During the first two years of the master's program, they participate in basic biomedical science courses alongside the university's medical students and take a seminar dedicated to issues in translational medicine. In the second year, they choose thesis labs and clinical mentors.	Provide masters level medical concept training for PhD students
**MD-PhD Resources**	Association of American Medical Colleges http://tinyurl.com/6wwa9eh	Provides links to resources related to MD/PhD training, including MD-PhD training programs in the United States and Canada and workshops and research programs for students interested in pursuing combined MD-PhD training.	Provide information about MD-PhD training
**Med into Grad Initiative**	Howard Hughes Medical Institute http://tinyurl.com/7n7szu3	Supports and encourages graduate schools to integrate medical knowledge into their PhD training. The program's goal is to produce researchers who will recognize which biological problems are of the greatest clinical relevance, have the knowledge and skills necessary to facilitate the translation of new biological knowledge into tools to improve human health, and be able to create fruitful research partnerships with physicians.	Integrate medical knowledge into PhD training
**National CTSA Educational Resource Program**	University of Rochester http://tinyurl.com/cqkgyhc	Makes available a variety of clinical and translational educational modules from the CTSA consortium training programs and the NIH Institutes and Centers. The modules are specialized educational courses intended for audiences from K-12 students to experienced faculty and include a mix of materials for web- and classroom-based learning.	Educate and train students and investigators at all levels
**Physiology InFocus: Physiology in Medicine/ Translational Physiology Track**	The American Physiological Society http://tinyurl.com/7xopj9x; http://tinyurl.com/6wx2opr	Highlights translational research programming at the society's annual meeting through designated sessions and meeting tracks.	Educate graduate students, postdoctoral researchers, and independent investigators

**The Seminars in Translational Research (STRECH)**	University of Texas at San Antonio http://translationalseminars.utsa.edu/	Seminar series that brings together investigators from basic, clinical, and social sciences from multiple university campuses. Seminars highlight the translation of research discoveries from bench to bedside to community and promote the formation of multidisciplinary clinical and translational research teams.	Promote education and collaboration
**Translational Medical Research Symposia Series and Workshop**	American Federation for Medical Research http://www.afmr.org/eb2011.cgi	Annual researcher-initiated and peer-reviewed seminar series showcasing bench-to-bedside research addressing cutting edge, clinically important topics. AFMR also conducts a workshop to provide investigators with perspectives and practical tools for developing successful clinical-translational medical research programs. Workshops topics have included drug development, intellectual property, connecting research cultures, and managing conflict of interest.	Educate graduate students, postdoctoral researchers, and independent investigators
**Translational Cancer Research for Basic Scientists**	American Association for Cancer Research http://tinyurl.com/4xhtcsy	Provided an intensive week-long introduction to translational cancer research for basic scientists transitioning to translational research. The goals were to provide participants a better understanding of translational research, teach them how to adapt their research for maximum clinical impact, and help them transition into a new career in translational cancer medicine.	Educate graduate students, postdoctoral researchers, and independent investigators
**TRIUMPH (Translational Research in Multi-Disciplinary Program) Postdoctoral Fellowship**	University of Texas (UT) Graduate School of Biomedical Sciences, MD Anderson Cancer Center, UT Medical School http://tinyurl.com/3fprqhk	Provides training in clinical and translational research to postdoctoral researchers through didactic course work, clinical rotations, and mentorship. The long-term goal is to produce translational scientists who can be teamed with suitable physician scientists in translational research laboratories.	Educate and train postdoctoral researchers

**Recognizing and Rewarding Translational Research**			
**Resource, Policy, or Program**	**Organization**	**Description**	**Purpose**
**Team Science Award**	American Association for Cancer Research http://tinyurl.com/38ha78b	Recognizes an outstanding interdisciplinary research team for innovative and meritorious science that has or will likely advance fundamental knowledge of cancer or that has applied existing knowledge to advance the detection, diagnosis, prevention, or treatment of cancer. Teams are composed of independent faculty researchers with complementary interdisciplinary expertise, each of whom has made separate substantive and quantifiable contributions to the research.	Recognize team scientists
**Tenure Track Offer Letter Template: Team and Interdisciplinary Science**	National Institutes of Health http://tinyurl.com/c38kmu9	Provides guidelines for writing letters of offer to, and reviewing and rewarding, interdisciplinary scientists. It includes a set of questions to help identify the roles, responsibilities, and expectations of scientists and their departments.	Provide guidance on appointing interdisciplinary, collaborative researchers
**University of Southern California Tenure and Promotion Manual**	University of California http://tinyurl.com/c2gehxb	Provides a model for how academic departments and committees can weigh interdisciplinary research and collaborative scholarship when making tenure and promotion decisions.	Provide guidance on rewarding interdisciplinary, collaborative research

**General Resources**			
**Resource, Policy, or Program**	**Organization (weblink)**	**Description**	**Purpose**
**CTSA Consortium's Resources for Researchers**	CTSA Consortium http://tinyurl.com/6peeoab	Provides links to information for basic, clinical, and translational research communities, including research networks, research tools, and funding opportunities available through public and private institutions.	Identify research resources

**Team Science Toolkit**	National Cancer Institute (NIH) https://www.teamsciencetoolkit.cancer.gov	Provides a user-generated collection of information and resources that support the practice and study of team science. It connects professionals from many disciplines and provides a forum for sharing knowledge and tools.	Identify research resources

**Translational Research Funding**		
**Organization**	**Description**	**Web Link**
**National Institute of Health**	Provides translational research support for basic investigators through investigator-initiated and targeted grants programs for individuals and institutions, as well as opportunities to access resources, instrumentation, and core facilities. Specific programs aimed at facilitating translational science are described in the main text (Box A).	http://nih.gov/
**U.S. Department of Veterans Affairs**	Provides translational research support for basic and clinical VA investigators through investigator-initiated merit review awards and targeted announcements. Investigators can also access translational research resources such as the Clinical Research Pharmacy and Pharmacogenomics Analysis Laboratory. Additionally, the VA supports several relevant career development programs, including the Career Development Enhancement Award, which provides support for educational leave to learn new research skills.	http://www.research.va.gov/
**Department of Defense**	Provides translational research support to intramural and extramural basic investigators. Extramural programs include the Congressionally Directed Medical Research Programs, Defense Advanced Research Projects Agency, and the Defense Medical Research and Development Program.	http://www.defense.gov/ http://www.darpa.mil/ http://cdmrp.army.mil/ http://dmrdp.fhpr.osd.mil
**Nongovernmental, Not-for-profit organizations**	Provides grant funding for translational researchers and research teams. Many, but not all, of these programs are targeted at specific diseases, conditions, or biological systems. The Health Research Alliance (see link) is a consortium of 50 such organizations, some of which have translational research funding opportunities for basic scientists.	http://www.healthra.org/
